# A predictive model of immune infiltration and prognosis of head and neck squamous cell carcinoma based on cell adhesion-related genes: including molecular biological validation

**DOI:** 10.3389/fimmu.2023.1190678

**Published:** 2023-08-24

**Authors:** Yuchen Liu, Zhechen Wu, Ziyue Fu, Yanxun Han, Jianpeng Wang, Yanqiang Zhang, Bingyu Liang, Ye Tao, Yuchen Zhang, Chuanlu Shen, Yidan Xu, Siyue Yin, Bangjie Chen, Yehai Liu, Haifeng Pan, Zhang Liang, Kaile Wu

**Affiliations:** ^1^ Department of Otolaryngology, Head and Neck Surgery, The First Affiliated Hospital of Anhui Medical University, Hefei, Anhui, China; ^2^ Anhui Medical University, Hefei, Anhui, China; ^3^ Department of Oncology, The First Affiliated Hospital of Anhui Medical University, Hefei, Anhui, China; ^4^ Department of Epidemiology and Biostatistics, School of Public Health, Anhui Medical University, Hefei, Anhui, China

**Keywords:** head and neck squamous cell carcinoma, focal adhesion-related gene, prognosis, immune infiltration, chemotherapy sensitivity

## Abstract

**Background:**

Focal adhesion serves as a bridge between tumour cells and the extracellular matrix (ECM) and has multiple roles in tumour invasion, migration, and therapeutic resistance. However, studies on focal adhesion-related genes (FARGs) in head and neck squamous cell carcinoma (HNSCC) are limited.

**Methods:**

Data on HNSCC samples were obtained from The Cancer Genome Atlas and GSE41613 datasets, and 199 FARGs were obtained from the Molecular Signatures database. The integrated datasets’ dimensions were reduced by the use of cluster analysis, which was also used to classify patients with HNSCC into subclusters. A FARG signature model was developed and utilized to calculate each patient’s risk score using least extreme shrinkage and selection operator regression analysis. The risk score was done to quantify the subgroups of all patients. We evaluated the model’s value for prognostic prediction, immune infiltration status, and therapeutic response in HNSCC. Preliminary molecular and biological experiments were performed to verify these results.

**Results:**

Two different HNSCC molecular subtypes were identified according to FARGs, and patients with C2 had a shorter overall survival (OS) than those with C1. We constructed an FARG signature comprising nine genes. We constructed a FARG signature consisting of nine genes. Patients with higher risk scores calculated from the FARG signature had a lower OS, and the FARG signature was considered an independent prognostic factor for HNSCC in univariate and multivariate analyses. FARGs are associated with immune cell invasion, gene mutation status, and chemosensitivity. Finally, we observed an abnormal overexpression of MAPK9 in HNSCC tissues, and MAPK9 knockdown greatly impeded the proliferation, migration, and invasion of HNSCC cells.

**Conclusion:**

The FARG signature can provide reliable prognostic prediction for patients with HNSCC. Apart from that, the genes in this model were related to immune invasion, gene mutation status, and chemosensitivity, which may provide new ideas for targeted therapies for HNSCC.

## 1 Introduction

In the United States, head and neck squamous cell carcinoma (HNSCC), which develops in the mucosal epithelia of the mouth, throat, and larynx, is the sixth most common cancer (1). Annually, around 900,000 cases and more than 400,000 fatalities from HNSCC are reported globally (2). Human papillomavirus (HPVs) infection, alcohol, and smoking consumption are risk factors associated with HNSCC (3). As a highly heterogeneous malignant tumour, the main reasons for high mortality are late-stage diagnosis, multiple anatomical sites, and diverse molecular characteristics (4). Standard treatments for HNSCC are surgery, radiation, and chemotherapy; however, the success rate of chemoradiotherapy for locally advanced diseases is relatively low (5). Clinicopathological staging systems have been used to predict the prognosis and guide treatment. However, owing to heterogeneity and particular biological characteristics, patients receiving the same stage and treatment may experience different results. Therefore, new biomarkers are urgently needed to assist clinicians in evaluating the prognosis and weighing the benefits and drawbacks of treatment decisions for patients.

Focal adhesion, the contact site between cells and the extracellular matrix (ECM), maintains the stability of cell tension and signaling for cell survival (6, 7). The reduction of focal adhesion molecule expression enhances epithelial-mesenchymal transition, which is a prerequisite for tumour cell invasion and metastasis (8, 9). Integrins, focal adhesion kinases, and growth factor receptors are focal adhesion molecules linked to cancer progression (10). Considering their most upstream distribution, receptors within focal adhesions are a good starting point for therapeutic intervention (11). Therefore, focal adhesion-related genes (FARGs) are potential prognostic indicators and targets for therapies in HNSCC patients.

Immune checkpoint inhibitors (ICIs) are promising anti-tumor immunotherapy, which has been authorized by the FDA for the management of advanced HNSCC, with significant efficacy in some patients. But after getting ICI treatment, some patients develop resistance to drugs and disease progression, which may be due to tumours with infiltrated immune cells (12, 13). Recently, cell adhesion to ECM has been recognised as a significant predictor of cancer cell resistance to several microenvironmental variables (11). Therefore, a better understanding of the tumour immune microenvironment (TIME) and FARGs may contribute to improving the sensitivity of HNSCC patients to ICI therapy.

Overall, a FARG signature was constructed using integrative analysis, which is a useful predictive indicator for patients with HNSCC. Furthermore, we observed links between the FARG risk score and tumour mutation frequency, immune invasion, chemosensitivity, and immunotherapy sensitivity, which may provide effective treatment guidance for patients with HNSCC.

## 2 Materials and methods

### 2.1 Data collection and procession

The Cancer Genome Atlas (TCGA) gene expression and clinical data for HNSCC can be retrieved using the GDC portal (https://portal.gdc.cancer.gov).The Gene Expression Omnibus (GEO, http://www.ncbi.nlm.nih.gov/geo/) provided the GSE41613 chips. We obtained 199 FARGs from the Molecular Signatures database (MSigDB; https://www.gsea-msigdb.org/gsea/msigdb). The focal adhesion-relevant genes are shown in **Supplement Table 1**.

### 2.2 Tumor classification and subtypes analysis

Prognosis-correlated FARGs were analysed using univariate Cox regression analysis (P < 0.05). HNSCC samples were categorised into different subtypes using consensus non-negative matrix factorisation (CNMF) approaches. To validate and assess the differences between the tumour subtypes, we first took a survival analysis. The expression of prognostic FARGs differed between the two subtypes. For TIME, we used the CIBERSORT, ssGSEA, and MCPcounter algorithms to quantify the expression of immune cells and then compared immune scores and immune cell numbers across distinct tumour subtypes. Human leukocyte antigen (HLA) mRNA expression was measured and compared for each sample. In addition, hallmark gene sets and Gene Set Variation Analysis (GSVA) were applied to detect enrichment levels of each pathway in both subgroups.

### 2.3 Construction and validation of HNSCC prognostic feature based on focal adhesion-related genes

We used LASSO-Cox regression analysis on the TCGA cohort to identify critical genes that were closely related to overall survival (OS). Subsequently, the following formula was applied for getting FARG’s risk score: score = ∑ (exp_i_ × correspondence coefficient of gene i). Using the FARG risk scores, we then divided the patients into two groups: low-risk and high-risk. To evaluate and compare the survival differences between low-risk and high-risk groups, “survival” and “survminer” R packages were used. Receiver-operating characteristic (ROC) curves were employed to assess the prognostic ability of models. This operation was repeated on the GSE41613 dataset to verify the signature stability.

### 2.4 Construction and evaluation of nomogram

To identify independent prognosis-related factors, the risk score and other clinical features (sex, age, grade, T, N, and stage) were analysed using Cox regression analysis. Then, the “rms” package was applied to build a nomogram model, which combined risk scores and clinicopathological parameters with independent prognostic values. We developed and evaluated the calibration curve and concordance index (C-index) of the nomogram for the purpose to assess the performance of the models. The nomogram’s usefulness for therapy was further evaluated using decision curve analysis (DCA).

### 2.5 Association analysis of risk score and therapy sensitivity

The therapeutic responses to ICI therapy were predicted using the TIDE algorithm (http://tide.dfci.harvard.edu/) and Subclass Mapping (SubMap; https://cloud.genepattern.org/gp/). To observe the difference in the RNA levels of Programmed cell Death-Ligand 1(PD-L1) and Cytotoxic T Lymphocyte-Associated Antigen-4(CTLA-4) in the high- and low-risk groups, we obtained RNA expression levels of PD-L1 and CTLA-4 in the TCGA-HNSC dataset and used the R package ‘ ggpubr ‘ to create the box plot.

By creating a ridge regression model based on The Genomics of Drug Sensitivity in Cancer (GDSC, https://www.cancerrxgene.org) database, the program “pRRophetic” served for estimating the chemotherapeutic medicines sensitivity of all HNSCC samples in the TCGA cohort. The sensitivity variations for nine drugs, including cisplatin, methotrexate, paclitaxel, rapamycin, and mitomycin, were evaluated in the high- and low-risk groups. C: docetaxel, lapatinib, gemcitabine, and bleomycin. Additionally, we used drug sensitivity data from the CellMiner database (https://discover.nci.nih.gov/cellminer/home). do) to explore the relationships among hub prognosis, FARGs expression, and drug sensitivity.

### 2.6 Genes expression and mutation analysis

In TCGA database, the expression levels of nine genes in both normal and malignant tissues was examined. Copy number variation (CNV) frequency was computed. The “maftools” package and cBioPortal online database were used to investigate the mutational landscape.

### 2.7 Pathway and functions enrichment analysis

Differences in pathways and functions in the high-risk and low-risk groups were analysed by Gene Set Enrichment Analysis (GSEA) using Gene Ontology (GO), Kyoto Encyclopedia of Genes and Genomes (KEGG), hallmark, and reactome gene sets. Using GSVA, we additionally explored the connections between the expression of FARG and other pathways.

### 2.8 Relationship between risk score and tumor immune microenvironment

We used well-established approaches, including XCELL, TIMER, QUANTISEQ, MCPCOUNTER, EPIC, CIBERSORT-ABS, and CIBERSORT, to measure the degree of immune invasion in TCGA samples. Spearman’s correlation analysis and the Wilcoxon test were used to search for variations in immune content between the high-risk and low-risk groups. In addition, the association between risk scores, estimate scores, immune scores, stromal scores, and tumour purity was studied.

### 2.9 Real time PCR assay

The First Affiliated Hospital of Anhui Medical University provided HNSCC and surrounding non-cancerous tissues for this investigation. The ethics committee provided approval for the project. The participants included in this study were patients with squamous cell carcinoma of the head and neck at the Department of Otolaryngology, First Affiliated Hospital, Anhui Medical University. An experienced pathologist collected the tumor tissue and most distal mucosal tissue from the patient with HNSCC beyond the safe margin of the freshly resected surgical resection. Postoperative pathology analysis confirmed the absence of cancer cell infiltration in our experimental control tissue specimens. This procedure was performed clinically in strict accordance with the NCCN Guidelines® Insights: Head and Neck Cancers, Version 1.2022 and defined a safe cut margin as a margin 5 mm or more from the front of the cancer infiltrate (14).

From HNSCC samples and surrounding noncancerous tissues, total RNA was extracted using the TRIzol reagent, and cDNA was created using a reverse transcription kit (Evo M-MLV RT Premix, Thermo Fisher Scientific). SYBR ® Green Premix Pro Taq HS qPCR Kit (Accurate Biology) was used to perform RT-PCR on the synthesised cDNA. After the reaction, use the 2 − ΔΔ Ct method to process the data. As an endogenous control, GAPDH was utilized. **Supplementary Table 2** includes a list of the primers used.

### 2.10 Western blot

Precooled radioimmunoprecipitation assay buffer and phenylmethylsulphonyl fluoride solutions were mixed in a ratio of 100:1. The tissue was fragmented with the mixed solution. The obtained lysate was treated in a tissue grinder and centrifuged at 4°C at 12000 rpm for 30 min to remove the tissue fragments. The supernatant was separated and a quarter of the protein loading buffer was added. This mixture was boiled for 10 min at 100 °C. Next, the proteins were separated using PAGE Gel Fast Preparation Kit (Epizyme, Shanghai, China) and transferred to a polyvinylidene fluoride membrane. The membrane was incubated with primary antibodies at 4°C overnight. The membrane was immersed with the secondary antibody for 2 hours at room temperature after being washed 3 times in Tris-buffered saline with 0.1% Tween® 20. The visualization of protein bands was done using enhanced chemiluminescence. The ImageJ software was used to quantify and compare the grey density of the protein bands.

### 2.11 Immunohistochemistry

Formalin-fixed, paraffin-embedded tissue sections were dewaxed in dimethylbenzene and rehydrated with ethanol at a gradient concentration. Endogenous peroxidase activity was inhibited using 0.35% H2O2 in phosphate-buffered saline (PBS). The slices were sealed using 5% bovine serum albumin solution, treated with primary antibody, and incubated overnight at 4 °C. Goat anti rabbit IgG labelled with horseradish peroxidase was then added to the sections and incubated in 37° water bath for 30 min. The sections were then rinsed with PBS. Finally, the staining was observed and photographs were acquired using an Olympus CX41 fluorescence microscope (EVIDENT CORPORATION, Shinjuku-ku, Tokyo, Japan).

### 2.12 Cell culture and lentiviral infections

Human HNSCC cell lines (FaDu and CAL27) were obtained from the American Type Culture Collection (Manassas, VA, USA) and cultured in Roswell Park Memorial Institute (RPMI)1640 medium containing 10% foetal bovine serum (FBS). Cells were seeded in 6-well plates and cultured at 37°C and 5% CO_2_ in a cell culture incubator till the cell confluence reached 30–40% for lentiviral infection. The cells were stably transfected with short hairpin RNAs targeting MAPK9 using a lentiviral vector. The target sequences are listed in **Supplementary Table 3**. Two days after infection, the cells were selected by adding 2 µg/mL puromycin for 1 week, and no significant dead floating cells were observed under a microscope. Western blotting was used to validate the effectiveness of the transfection.

### 2.13 Cell counting kit-8

Cells were seeded onto 96-well plates with indicated treatment at 1000 cells/well, and incubated at 37°C and 5% CO_2_ in a cell incubator. Ten microlitres of CCK-8 solution was added to each well at specified time points, incubation was continued under the same conditions, and absorbance values were measured at 450 nm.

### 2.14 Colony formation assays

Approximately 1500 cells were inoculated into 60 mm plates and cultured at 37°C and 5% CO_2_ in a cell incubator. When the formation of cell clones in the plates was observed under a microscope and the size and number of clones were appropriate, the culture plates were washed with PBS. A 4% polymethanol fixative was added to each plate for 20 min, and then the fixative was discarded. The Giemsa staining solution was added, and the staining solution was removed with tap water after 30 min, dried, photographed, and the number of clones was calculated.

### 2.15 Wound healing assays

Cells were inoculated into 6-well plates, and when the cells were fully grown into a monolayer, cell scribing was performed. Using a straightedge as an aid, a vertical line was drawn using a 200 µL pipette tip. The culture medium was discarded, and the cells were washed three times using PBS to remove scratched cells. Serum-free medium was added to each well, and the cells were cultured in the cell incubator, observed, and photographed at 0 h and24 h under a 10x microscope.

### 2.16 Transwell experiment

The Transwell chambers were immersed in 75% ethanol 12 h in advance, rinsed with PBS, and irradiated with UV light for 2h to achieve sterility. The transwell chambers were placed in 24-well plates. The serum-free medium and matrix gel were mixed well at a ratio of 8:1, and 60 µL of the mixture was added to each transwell chamber, and then, the 24-well plate containing the transwell chambers was placed in the cell culture incubator overnight. Approximately 2×10^4^ cells in 200 µL serum-free medium were seeded in the upper chamber, and 500 µL of medium containing 10% FBS was added to the lower chamber. A 24-well plate with transwell chambers was placed back into the cell culture incubator and incubated for 24 h. Cells on the lower membrane surface were fixed in 4% formaldehyde for 15 min and stained with crystal violet for 30 min. The cultures were then rinsed with PBS, the excess staining solution was removed from the transwell chambers’ surface using a cotton ball, and the cells were counted under a microscope.

### 2.17 Statistical analysis

R (version 4.1.3) and GraphPad Prism 9.0 was applied for statistical analysis. LASSO Cox regression analyses were performed to identify prognosis-related genes. Kaplan–Meier (K-M) analysis and log-rank tests were used to perform the survival studies. The Wilcoxon test was employed for comparing the two groups, and Spearman’s correlation analysis was utilized for investigating connections.

## 3 Results

### 3.1 Eighteen prognostic genes and two tumor subtypes

**Figure 1:**
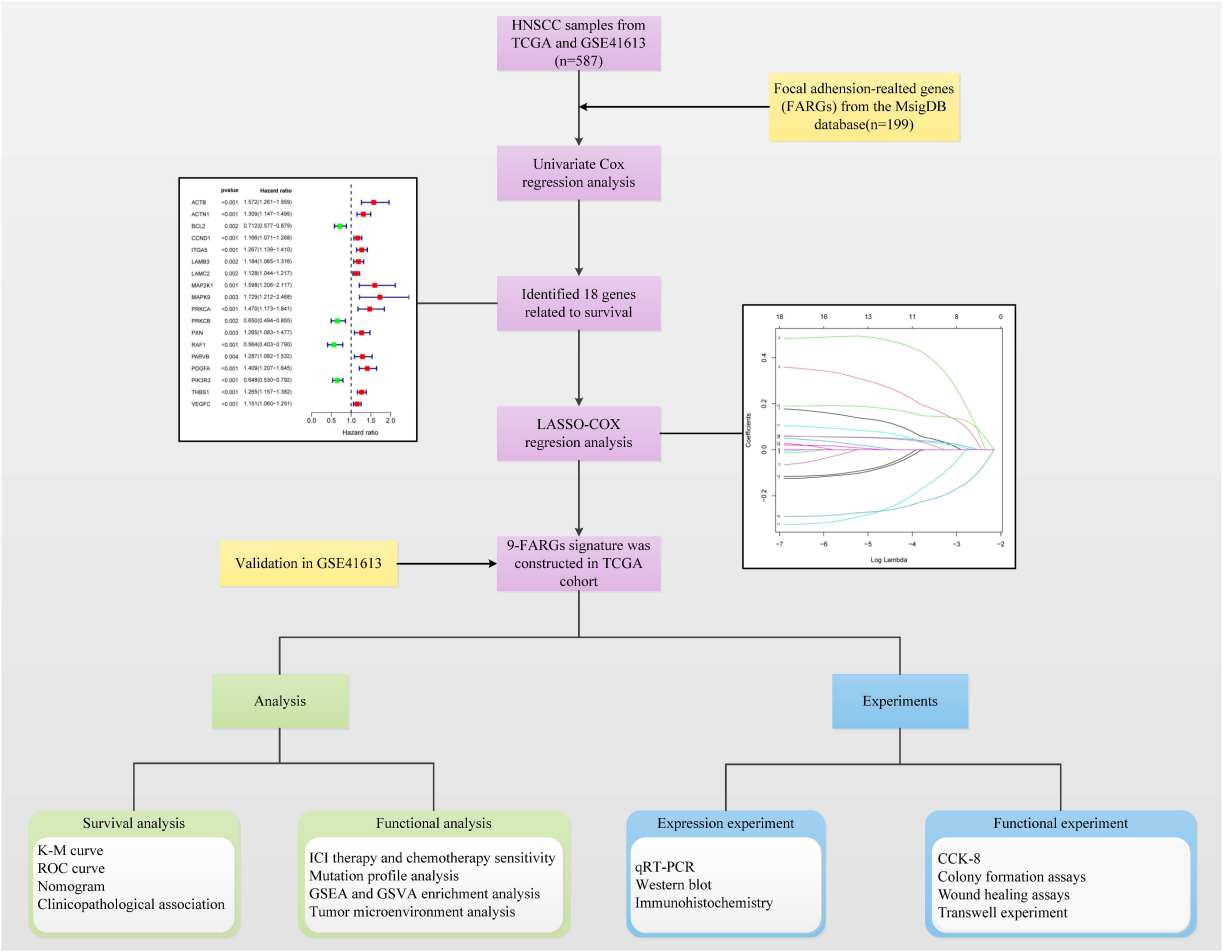
Flow chart of the study.

**Figure 2:**
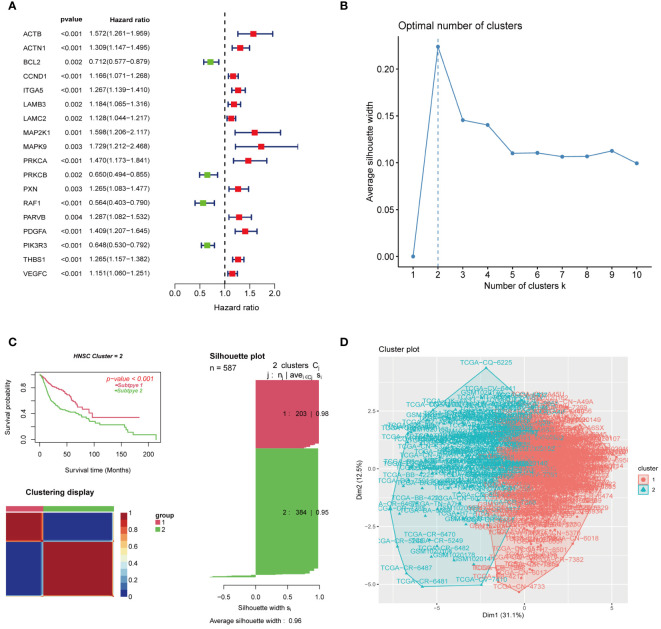
Gene screening and clustering analysis of focal adhesion-related genes. **(A)** Univariate regression analysis screened 18 genes associated with tumor prognosis. **(B)** K = 2 was the optimal number of subgroups using the K-means method. **(C)** The consensus non-negative matrix decomposition divided the samples into two HNSCC clusters, with significant differences in survival between the two clusters. The right side is the silhouette plot. When k = 2, the average silhouette width is the largest and the width is close to 1. This is consistent with the original grouping, which further proves the rationality of the results. **(D)** The classification plot divided the tumor samples into two tumor subtypes, which was the same as the classification result in **(C)**.

**Figure 1** shows the flow chart of this study. After excluding samples with a duration of survival of less than thirty days, we collected data from 587 HNSCC patients (TCGA:491 samples; GSE41613:96 samples). Univariate regression analysis of the genes from the database showed that eighteen genes were associated with tumour prognosis. BCL2, PRKCB, RAF1, and PIK3R3 are protective genes linked to a favourable prognosis. The expression of the remaining risk genes was linked to poor prognosis (**Figure 2A**). We then used the K-means algorithm and CNMF for clustering analysis to identify tumour subtypes based on eighteen genes. We found that tumour subtype clustering was the most stable and appropriate when K = 2 (**Figure 2B**). Two subtypes of the 587 tumour samples were identified (C1 = 203 and C2 = 384). Kaplan–Meier analysis of tumour subtypes revealed a notable difference in the probability of survival for the two groups (P < 0.001), with C2 having a lower survival rate than C1. The right side shows the silhouette plot. When k = 2, the average silhouette width was the largest and the width was close to 1. This is consistent with the original grouping, further proving the rationality of the results. (**Figure 2C**). The classification plot also supported the results of the tumour subtype classification (**Figure 2D**).

### 3.2 Difference and immuno-infiltration analysis of tumor subtypes

**Figure 3:**
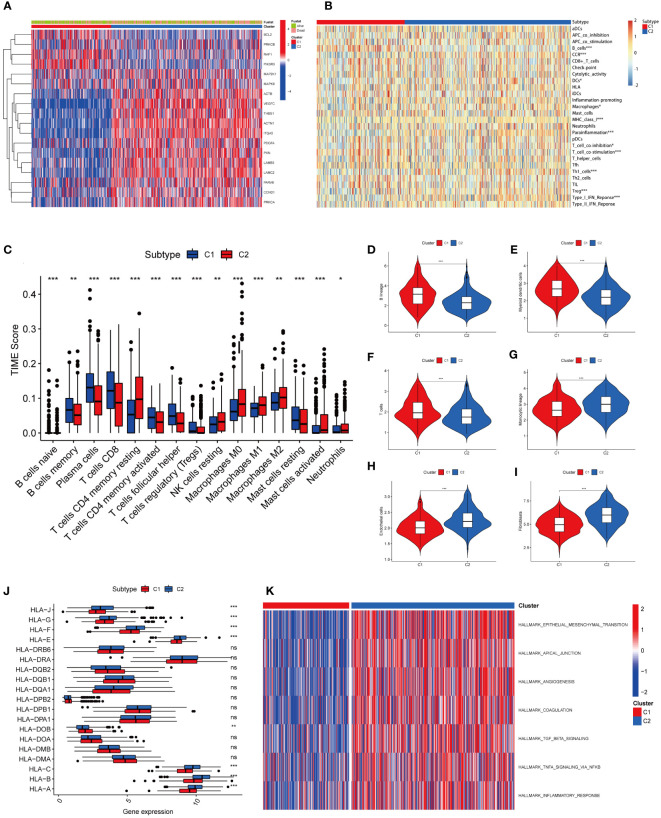
Heatmap results and immune infiltration analysis of two tumor subtypes. **(A)** The heatmap showed differences in prognostic gene expression between the two tumor subtypes. The difference was significantly correlated with survival or death status. **(B)** Heatmap showed different levels of immune cell expression in two tumor subtypes. **(C)** Abundance of 15 infiltrating immune cell types in two HNSCC subtypes. **(D)** Comparison of B lineage cells in two tumor subtypes. **(E)** Comparison of myeloid dendritic cells in two tumor subtypes. **(F)** Comparison of T cells in two tumor subtypes. **(G)** Comparison of monocytic lineage cells in two tumor subtypes. **(H)** Comparison of endothelial cells in two tumor subtypes. **(I)** Comparison of fibroblasts cells in two tumor subtypes. **(J)** The differences in HLA expression between the two tumor subtypes suggest that different tumor subtypes have different levels of ability to activate immune cells. **(K)** GSVA-based analysis of expression differences of pathways in two tumor clusters. (*represents P<0.05, **represents P<0.01, ***represents P<0.001; "ns" represents not significant. )

The heatmap revealed that prognostic genes were differentially expressed and strongly correlated with survival or death status between the two tumour subtypes. The expression of BCL2, PRKCB, RAF1, PIK3R3, and other genes related to prognostic improvement was high in C1, whereas the expression of ACTB, ACTN1, CCND1, ITGA5, LAMB3, LAMC2, MAP2K1, MAPK9, PRKCA, PXN, PARVB, PDGFA, THBS1, VEGFC, and other genes related to poor prognosis was high in C2. In addition, the patients in C2 had a higher death rate than those in C1 (**Figure 3A**). We utilized the CIBERSORT, MCP counter, and ssGSEA to measure gene expression of immune cells in each sample. The results showed that different tumour subtypes had different levels of immune cell expression and TIME (**Figures 3B–I**). B lineage (**Figure 3D**), myeloid dendritic cells (**Figure 3E**), and T cells (**Figure 3F**) were expressed significantly more in C1 than in C2; Endothelial cells (**Figure 3G**), fibroblasts (**Figure 3H**), and monocytes (**Figure 3I**) were expressed significantly more in C2 than in C1. In terms of HLA expression, HLA-A, HLA-B, HLA-C, HLA-E, HLA-F, HLA-G, and HLA-J were significantly more highly expressed in C2 than in C1, indicating that tumour subtypes differ in the activation of immune cells (**Figure 3J**). To analyze the connection between the enriched pathways and prognosis for HNSCC, we analysed the relative expression differences in the pathways of the two clusters using GSVA. The heatmap in **Figure 3K** shows that the two clusters differed significantly in epithelial mesenchymal transition and other pathways.

### 3.3 Construction and validation of 9 focal adhesion-related genes^’^ risk signature

**Figure 4:**
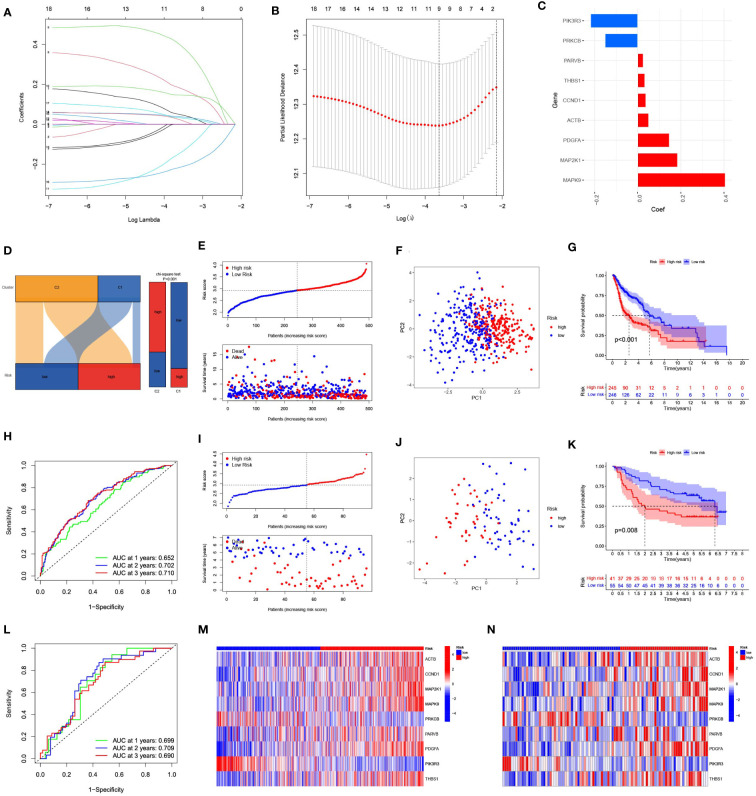
Construction and evaluation of a prognostic model based on 9 focal adhesion-related genes. **(A)** The LASSO coefficient profiles of 9 focal adhesion-related genes. **(B)** Tuning parameter (λ) selection cross validation error curve, the optimal log λ value is the left dotted line in the plot. **(C)** Construction of prognostic risk signature based on 9 genes. **(D)** The Sankey diagram on the left intuitively shows the relationship between tumor subtypes and risk groups. The bar chart on the right side shows the difference in the risk group composition of tumor subtypes calculated by chi-square test. **(E)** The distribution of risk score、survival time and OS status in TCGA. **(F)** The PCA results showed significant differences between the high-risk and low-risk groups. **(G)** The prognosis and survival time were significantly lower in the high-risk group than in the low-risk group. **(H)** ROC curve analysis of the 9 focal adhesion-related genes signature of the 1, 3, and 5 years in the TCGA. **(I–L)** Similar results were obtained using the same method in the GSE41613 dataset. **(M, N)** The heatmap shows that this has nine genes with significant differences in expression between the high and low risk groups in the TCGA **(M)** and GSE41613 **(N)** datasets.

Eighteen genes were subsequently examined using LASSO regression analysis (**Figures 4A, B**). Nine genes were used to create the prognostic risk signature, 9 genes were employed (**Figure 4C**). The expression patterns of the nine genes were multiplied by the relevant LASSO coefficients to calculate the risk score. The specific formula was as follows: Risk Score = (0.4058) × MAPK9 + (0.1848) × MAP2K1 + (0.1465) × PDGFA + (0.0507) × ACTB + (0.0379) × CCND1 + (0.0327) × THBS1 + (0.0251) × PARVB + (-0.1516) × PRKCB + (-0.2188) × PIK3R3. Using the median as the criterion, we separated all samples into high-risk and low-risk groups. The results revealed that C1 mainly included the low-risk groups, whereas C2 mainly included the high-risk groups (**Figure 4D**).

The risk score grew along with the mortality risk and survival time in the TCGA cohort (**Figure 4E**). Significant disparities between the high-risk and low-risk categories were shown by the PCA results, which also indicated feasibility and necessity of grouping (**Figure 4F**). The prognosis and time until death in the high-risk group were both significantly poorer than those in the low-risk group (**Figure 4G**). Using the ROC analysis, we obtained a satisfactory area under the curve (AUC). At 1, 3, and 5 years, the AUCs were 0.652, 0.702, and 0.710, respectively (**Figure 4H**). Similar results were obtained using the same approach in the GSE41613 cohort (**Figures 4I–L**). Nine genes exhibited substantial changes within the two groups based on an analysis of gene expression in the two groups, showing the same pattern in both cohorts (**Figures 4M, N**).

### 3.4 Association between risk signature and clinical features

**Figure 5:**
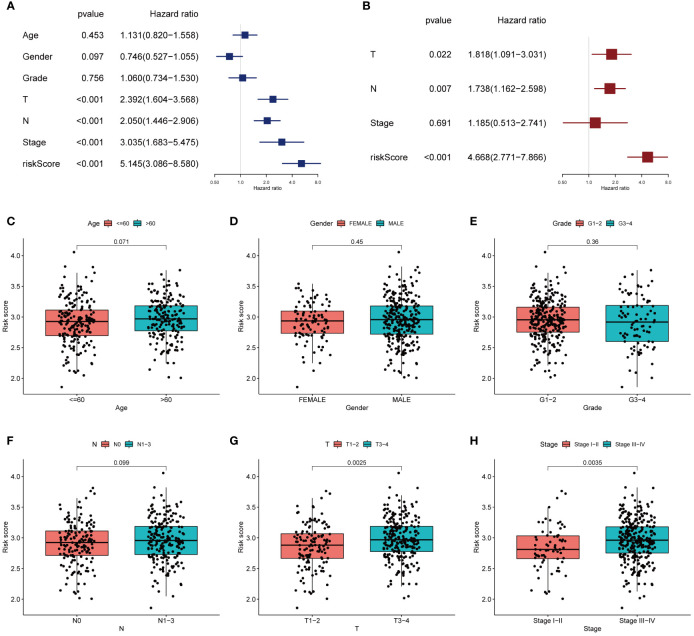
The correlations between the risk score and clinical factors. Univariate COX regression analysis **(A)** and multivariate COX regression analysis **(B)** showed that T stage, N stage and risk score were independent prognostic factors. **(C–H)** Correlation between risk score and age, gender, grade, N, T, and stage.

The T stage, N stage, and risk score were identified as independent prognosis-related factors using Cox regression analyses (**Figures 5A, B**). Subsequently, we analysed the spread of risk scores for various clinicopathological features (**Figures 5C–H**), and found that the risk score was higher for advanced T (**Figure 5G**) and stage (**Figure 5H**). We also divided the patients into subgroups according to their age, sex, grade, T, N, and stage to plot the K-M survival curve. In almost all subgroups, the OS of the high-risk group was shorter than that of the low-risk group (**Figures S1A–L**).

### 3.5 Construction and evaluation of nomogram

**Figure 6:**
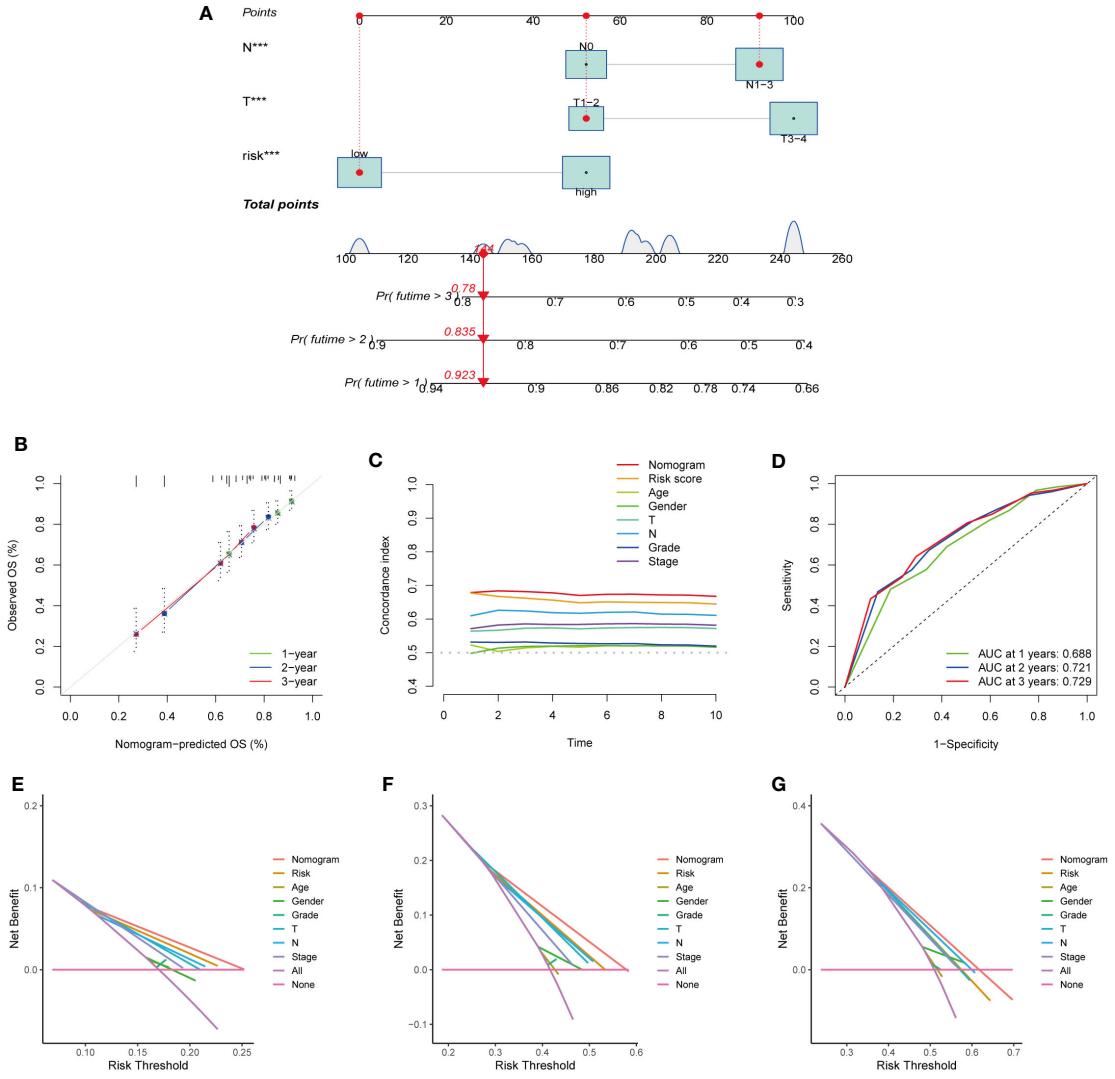
Establishment and evaluation of nomogram model in the TCGA dataset. **(A)** The nomogram for predicting the OS of patients at 1, 3, and 5 years. **(B)** Calibration curves of the nomogram for 1, 3, and 5 years. **(C)** The consistency index of nomogram was superior to each clinical factor, demonstrating the superiority of nomogram and signature. **(D)** ROC curve analysis showed the validity of the nomogram to predict the overall survival of patients at 1, 3 and 5 years. **(E–G)** Decision curve analysis at 1 **(E)**、3 **(F)**、and 5 **(G)** years. (***represents P<0.001).

To predict the 1-, 3-, and 5-year survival rates of patients with HNSCC, we created a nomogram that included clinical indicators (T and N) and risk scores (**Figure 6A**). The calibration plot displayed the discrepancy between the predicted and actual survival probabilities in patients with HNSCC (**Figure 6B**), indicating the nomogram’s high prediction accuracy. The C-index of the nomogram was higher than that of each element (**Figure 6C**), indicating the reliability of the nomogram as a predictor. The ROC curve revealed that the nomograms efficiently predicted the OS of patients efficiently (AUC = 0.688, 0.721, and 0.729) (**Figure 6D**). The DCA of the nomogram demonstrated that the model had excellent strength for 1-, 3-and 5-year OS (**Figures 6E–G**), which showed that the nomogram could serve as an efficient way to predict the prognosis of patients in clinical practice.

### 3.6 Risk scores predict the therapy sensitivity of HNSCC patients

**Figure 7:**
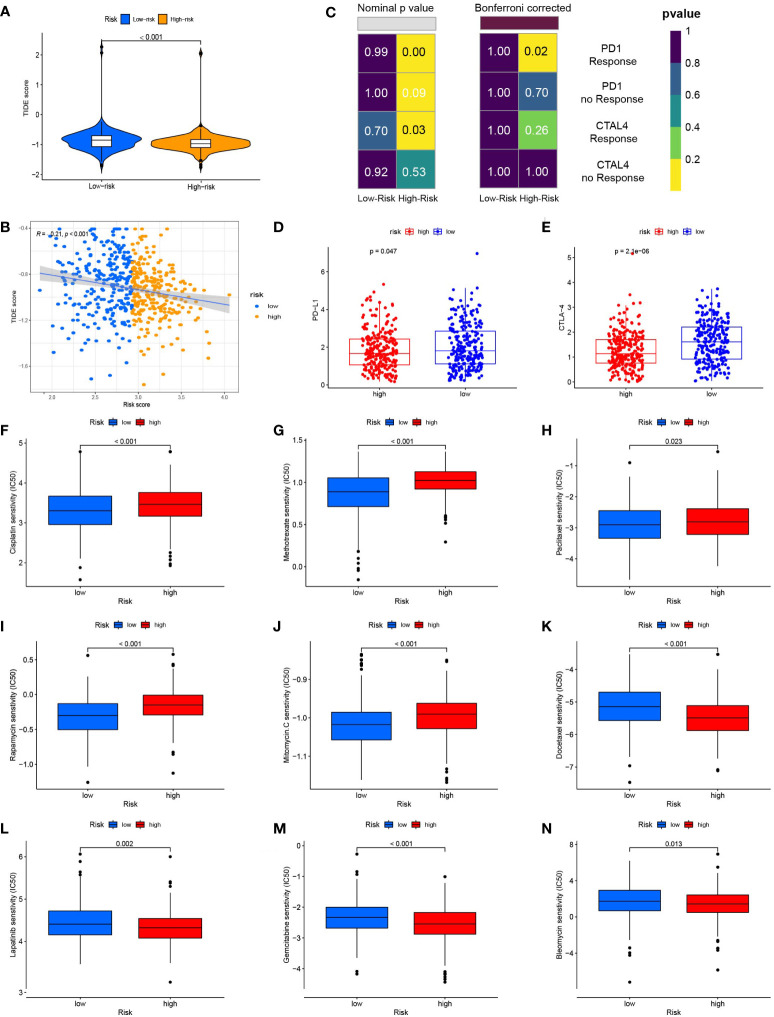
Risk score predicts responses to immunotherapy and chemotherapy. **(A)** TIDE scores were significantly lower in the high-risk group than in the low-risk group. **(B)** Risk scores were negatively correlated with TIDE scores. **(C)** Using the SubMAP algorithm, we inferred the possibility of anti-programmed cell death protein 1(PD1) and anti- cytotoxic T-lymphocyte-associated protein 4 (CTLA4) response immunotherapy in the high and low risk groups. High risk group may respond better to PD-1 treatment (Bonferroni-corrected P = 0.02). **(D)** The expression level of PD-L1 in the high and low risk groups **(E)** The expression level of CTLA-4 in high and low risk groups. **(F)** The box plot of the estimated IC50 for cisplatin. **(G)** The box plot of the estimated IC50 for methotrexate. **(H)** The box plot of the estimated IC50 for paclitaxel. **(I)** The box plot of the estimated IC50 for rapamycin. **(J)** The box plot of the estimated IC50 for mitomycin. C **(K)** The box plot of the estimated IC50 for docetaxel. **(L)** The box plot of the estimated IC50 for lapatinib. **(M)** The box plot of the estimated IC50 for gemcitabine. **(N)** The box plot of the estimated IC50 for bleomycin.

We used the TIDE algorithm to assess the likelihood of a response to immunotherapy. The findings revealed that the high-risk group’s TIDE score was much lower (**Figure 7A**), and the correlation within risk score and TIDE score was negative (**Figure 7B**), suggesting that the high-risk group might be more likely to benefit from immunotherapy. We also compared the expression profiles of TCGA-HNSCs with those of a separate cohort of 47 patients with melanoma who received immunotherapy using the SubMap algorithm. We found that anti-PD-1 antibody therapy had a higher chance of success in the high-risk group (nominal, p<0.001; Bonferroni-corrected, p=0.02) (**Figure 7C**). The RNA expression levels of PD-L1 and CTLA-4 were also consistent with this result, The expression levels of PD-L1 and CTLA-4 in the high-risk group were lower than those in the low-risk group (**Figures 7D, E**). High expression of PD-L1 and CTLA-4 inhibits tumour immunity and induces tumour immune escape.

**Figure 8:**
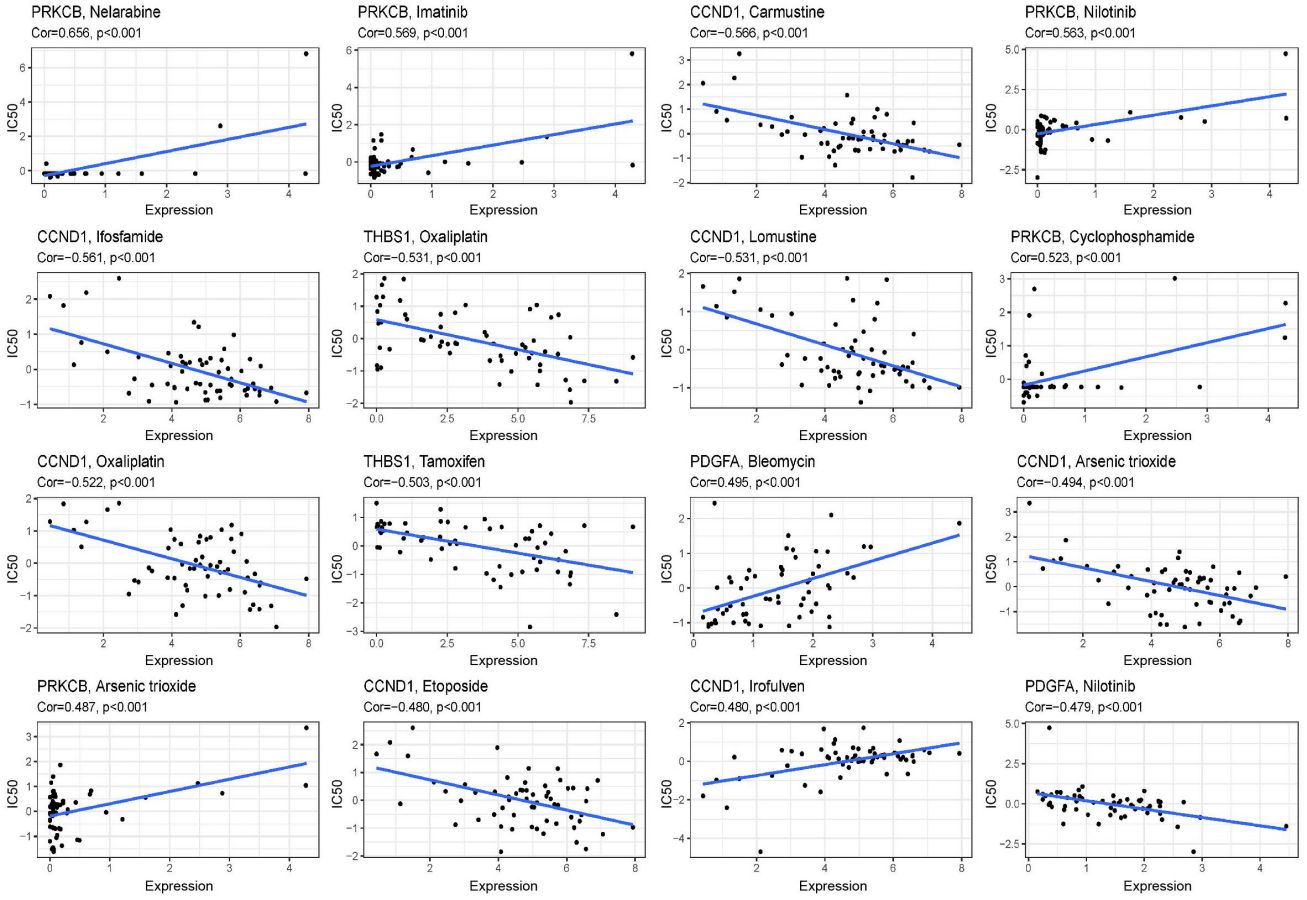
Scatter plots of the correlation between the expression of 9 genes and the predicted drug response. The vertical axis represents drug sensitivity, represented by Z-score, and the horizontal axis represents gene expression, represented by log2 (FPKM + 1).

We compared the sensitivity of the low-risk and high-risk groups to nine popular HNSCC chemotherapy medicines using the GDSC drug data. In patients with low FARG scores, the IC50 values of cisplatin (**Figure 7F**), methotrexate (**Figure 7G**), paclitaxel (**Figure 7H**), rapamycin (**Figure 7I**), and mitomycin were determined. Mitomycin.C (**Figure 7J**) were lower and more sensitive. The group at higher risk responded better to docetaxel (**Figure 7K**), lapatinib (**Figure 7L**), gemcitabine (**Figure 7M**), and bleomycin (**Figure 7N**). The correlation between 9 genes expression and the predicted drug response is shown in **Figure 8**. This implied that our FARG signature has important applications in clinical drug use.

### 3.7 Mutation spectrum of FARG signature in HNSCC

**Figure 9:**
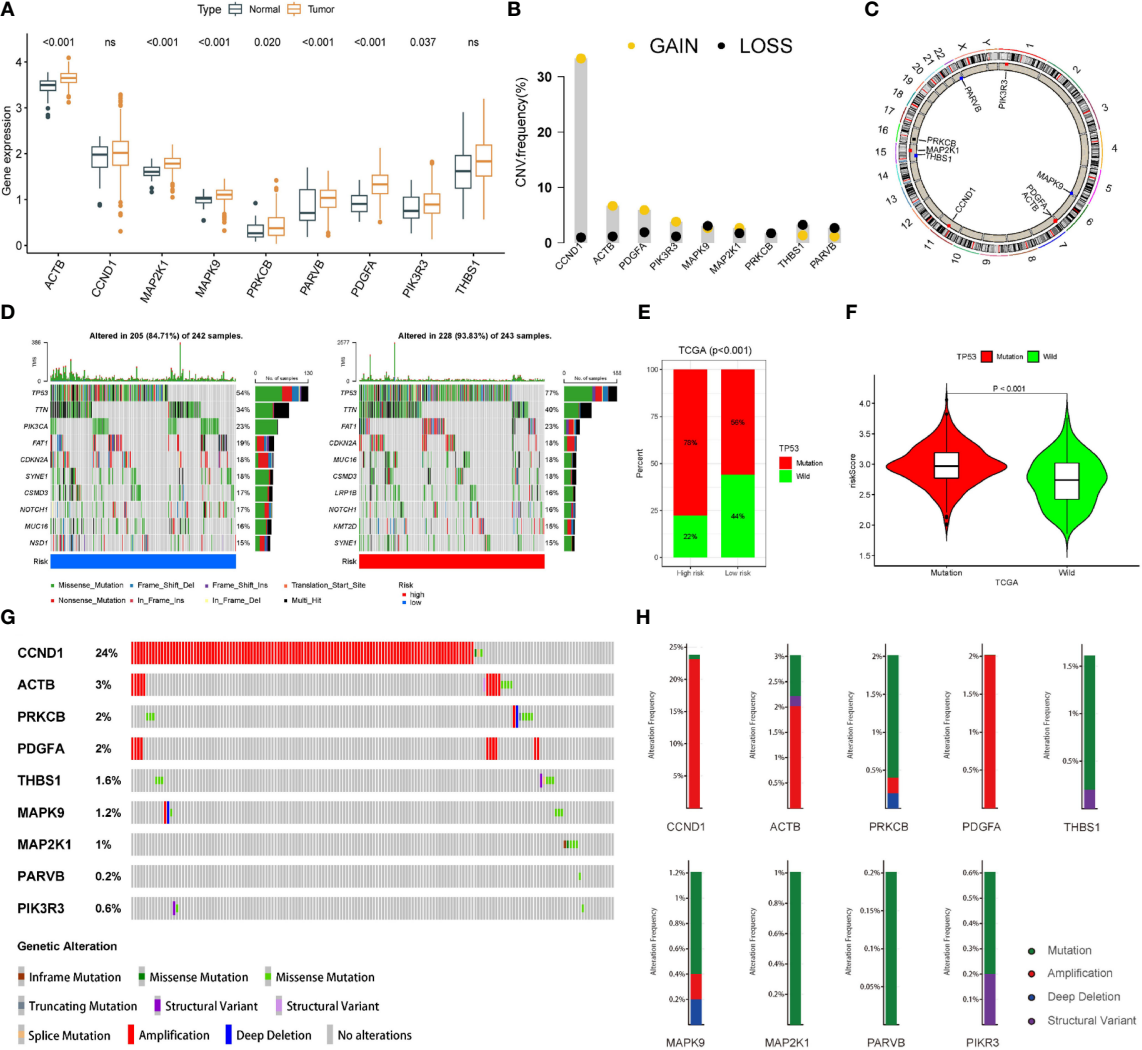
The landscape of genetic and expression variation of 9 focal adhesion-related genes in TCGA dataset. **(A)** Expression of 9 genes in tumor tissues. **(B)** The frequencies of CNV mutations in 9 genes. **(C)** The location of 9 focal adhesion-related genes on the chromosome. **(D)** Comparison of the top 10 mutated genes in the high-risk and low-risk groups. **(E)** Mutation difference of TP53 in high and low-risk groups. **(F)** The FARG score of patients with TP53 mutation was higher. **(G)** Alterations in each gene were assessed by analyzing the HNSCC samples in the cBioPortal database. **(H)** The detailed mutation spectrum of 9 focal adhesion-related genes.

In the TCGA-HNSC cohort, we found that ACTB, MAP2K1, PARVB, and PDGFA were upregulated in tumour tissues, suggesting that gene expression may be associated with tumourigenesis (**Figure 9A**). We also analysed the mutations in nine FARGs in patients with HNSCC. The frequency of CNV is summarised in **Figure 9B**, and chromosomal sites of these genes are shown in **Figure 9C**. We then performed gene mutation analysis according to the FARG signature and discovered a higher mutation count in the high-risk group than in the low-risk group (**Figure 9D**). Moreover, after the top 10 mutated genes for high- and low-risk samples were contradistinguished, we discovered that missense mutations were a particularly prevalent type of mutation, and in both groups, TP53 was the most commonly mutated gene (**Figure 9D**). We also found a larger percentage of patients with TP53 mutations in the high-risk group (**Figure 9E**) and the FARG scores of patients with TP53 mutations were higher (**Figure 9F**). In addition, we assessed the alterations in each gene by analysing HNSCC samples from the cBioPortal database. The total mutation frequency was 35.6%, and all genes in the FARG signature were mutated, indicating that these nine genes are crucial for the development of HNSCC (**Figure 9G**). The detailed mutation spectrum for each gene is shown in **Figure 9H**.

### 3.8 GSEA and GSVA analysis

**Figure 10:**
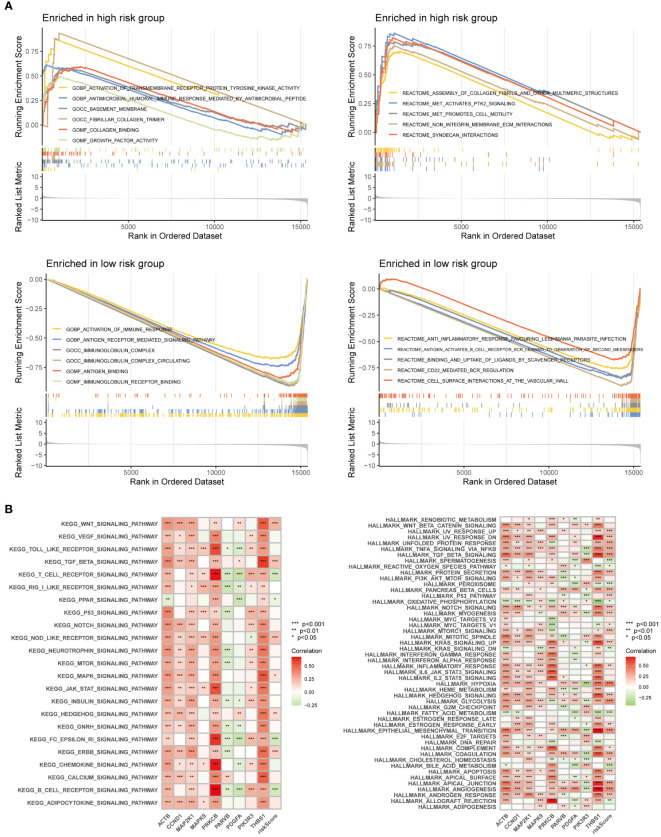
Enrichment analysis was performed based on GSEA algorithm and GSVA algorithm. **(A)** Differences in pathways and biological functions between the high and low-risk groups. **(B)** The results of KEGG and Hallmark analysis showed the correlation of genes and pathways.

GO and Reactome enrichment analyses based on GSEA indicated that multiple pathways and biological functions differed between the high- and low-risk groups (**Figure 10A**). KEGG and Hallmark enrichment analysis according to the GSVA algorithm showed the correlation of each gene and pathways (**Figure 10B**). These results may be due to the potential mechanism underlying the differences between the different FARG groups.

### 3.9 Relationship between risk score and tumour immune microenvironment

**Figure 11:**
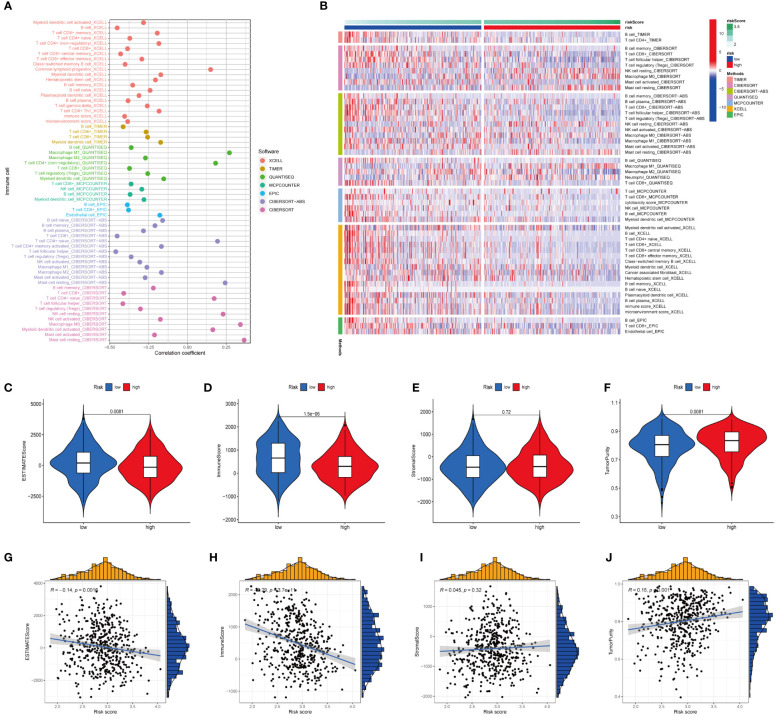
Analysis of immune landscape between the high-risk and low-risk groups in the TCGA dataset. **(A)** Association of risk scores with immune microenvironment as shown by Spearman correlation analysis. **(B)** Differences in immune cell abundance between the high-risk and low-risk groups. **(C)** Differences in ESTIMATE scores between the two groups. **(D)** Differences in immune score between the two groups. **(E)** Differences in stromal score between the two groups. **(F)** Differences in tumor purity between the two groups. **(G)** Risk scores were negatively correlated with ESTIMATE scores. **(H)** Risk scores were negatively correlated with immune score. **(I)** Risk score was positively correlated with stromal score. **(J)** Risk score was positively correlated with tumor purity.

To study the association between the FARG score and TIME, we used seven different applications for analysis. When utilizing Spearman’s correlation to investigate the relationship between the risk ratings and TIME (**Figure 11A**), CD8+ T cells, B cells, M2 macrophages, myeloid dendritic cells, and other types of immune cells were remarkably associated with the FARG score. The heatmap shows that the abundance of multiple immune cells varied greatly between the high- and low-risk groups (**Figure 11B**). The links between risk scores, estimate scores, immune scores, stromal scores, and tumour purity were studied (**Figures 11C–J**). The results suggested that the high-risk group’s estimation score (**Figure 11C**) and immune score (**Figure 11D**) were much lower than those of the low-risk group, but tumour purity (**Figure 11F**) was much higher. Moreover, correlation analysis revealed that the FARG score was strongly negatively linked with the estimated score (**Figure 11G**) and immune score (**Figures 11H**) and significantly positively correlated with tumour purity (**Figure 11J**).

### 3.10 Validation of hub genes mRNA and protein in cancer and paracancerous tissues

**Figure 12:**
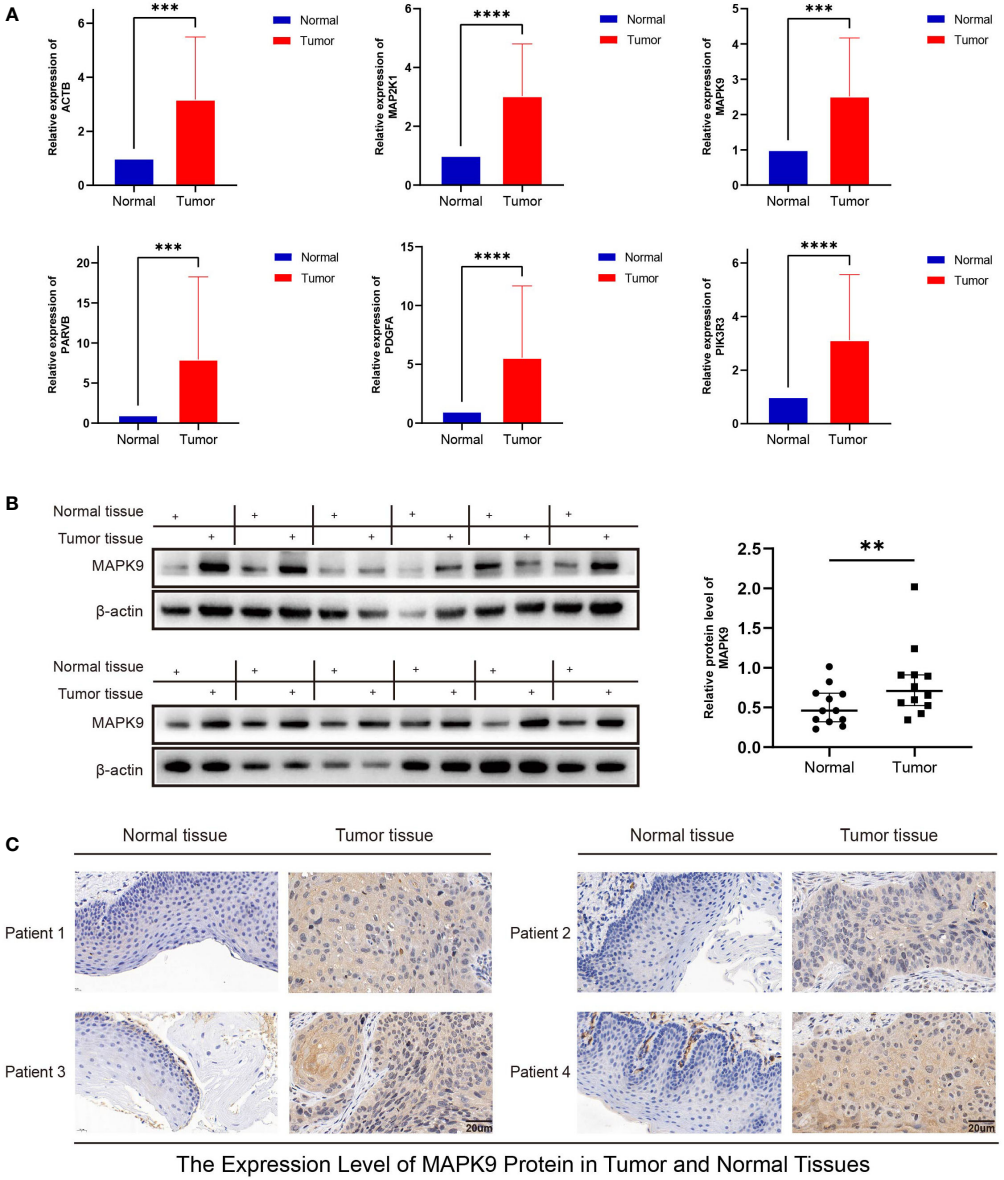
Validation of mRNA and protein expression levels of focal adhesion-related genes in HNSCC. **(A)** The expression of mRNA in HNSCC tissues and adjacent normal tissues was compared by RT-PCR. **(B)** The western blot analyses. **(C)** Immunohistochemical staining analysis. (**represents P<0.01, ***represents P<0.001, ****represents P<0.0001).

RT-PCR confirmed the expression of ACTB, MAP2K1, MAPK9, PARVB, PDGFA, and PIK3R3 mRNA in HNSC and adjacent tissues. The findings revealed that 12 cancer tissues exhibited higher expression of these genes than normal tissues. (**Figure 12A**). The expression of CCND1, PRKCB, or THBS1 showed no statistically significant difference (**Figure S2**), but the pattern matched the outcomes of the bioinformatics analysis. Western blotting and immunohistochemistry staining were used to confirm the MAPK9 protein expression level, and the results were consistent with the mRNA expression level (**Figures 12B, C**).

### 3.11 Knockdown of MAPK9 reduces the proliferation, migration and invasion of HNSCC cells

**Figure 13:**
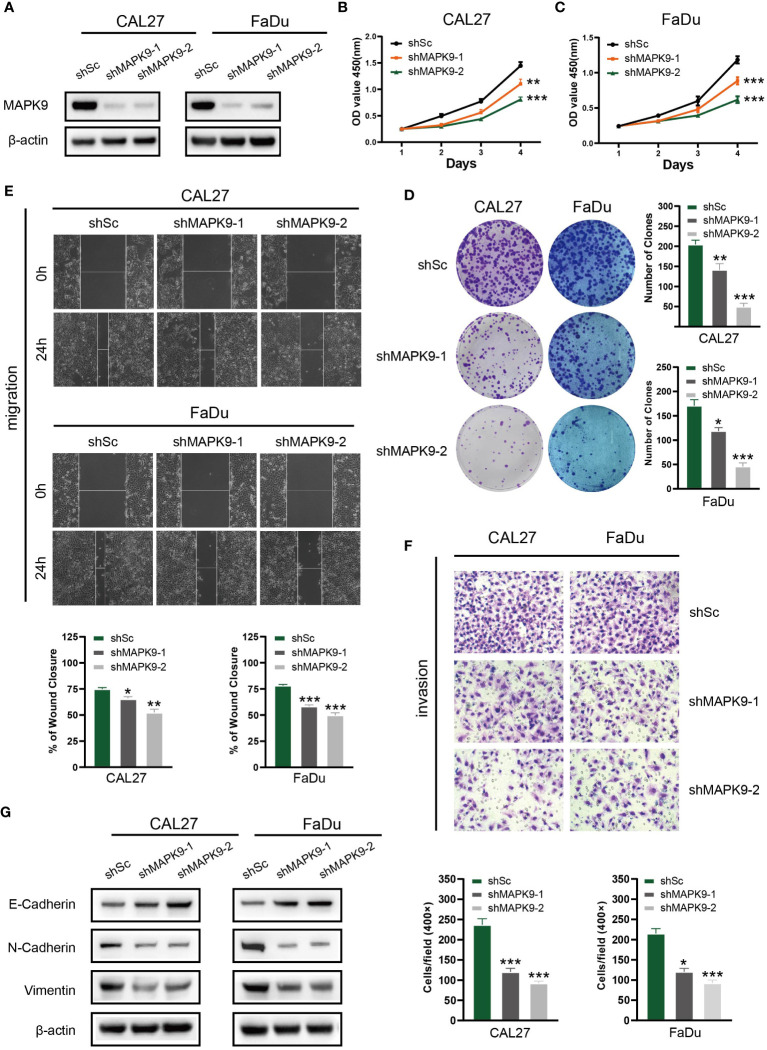
Knockdown of MAPK9 decreases HNSCC cells proliferation, migration and invasion. **(A)** Western blot validation of MAPK9 knockdown in CAL27 and FaDu cell lines. Cell growth rates, migration and invasion abilities of the indicated cells were evaluated by CCK-8 assays **(B, C)**, colony formation assays **(D)**, wound healing assays **(E)** and transwell **(F)**. Data were indicated as mean ± SD of triplicate technical replicates, *p < 0.05, **p < 0.01, ***p < 0.001 vs control group. **(G)** The expression of EMT markers (E-cadherin, N-cadherin and Vimentin) was detected by Western blot analysis when MAPK9 was knockdown in HNSCC cells.

To investigate possible biological functions and mechanisms of MAPK9 in HNSCC, we created stable MAPK9 knockdown HNSCC cell lines. The efficacy of cell transfection was verified using western blotting (**Figure 13A**). CCK-8 and colony formation tests were utilized to assess the proliferation capacity of HNSCC cells. The outcomes showed that MAPK9 knockdown greatly reduced the viability and colony formation ability of CAL27 and FaDu cells (**Figures 13B–D**). Moreover, we examined whether MAPK9 contributes to the migration and invasion of HNSCC cells. Wound healing and Transwell assays suggested that cell migration and invasion were markedly decreased after MAPK9 knockdown (**Figures 13E, F**).

Additionally, Western blot experiments showed that CAL27 and FaDu cells with MAPK9 knockdown had higher E-cadherin expression and lower expression of vimentin and N-cadherin (**Figure 13G**). These results indicate that MAPK9 encourages HNSCC cell migration by changing the expression of EMT-associated proteins. This also validated the results of **Figure 3K** in bioinformatics analysis.

## 4 Discussion

HNSCC is a heterogeneous tumour with a high incidence, insidious onset, and a lack of clear prognostic markers (15). Patients with HNSCC are diagnosed at an advanced or metastatic stage in 60% of cases (16). For patients with recurrent or metastatic HNSCC (R/MHNSCC), traditional treatment methods (surgery, radiotherapy, and chemotherapy) have poor prognosis and limited efficacy, with average duration of survival of only 11.6 months (17). Therefore, discovery of new prognostic biomarkers and treatment methods is a critical issue that needs to be addressed in clinical practice. Recent studies have shown that reduced expression of focal adhesions can promote epithelial-mesenchymal transition (EMT), which is a crucial basis for tumour cell invasion and metastasis (9, 18). FARGs have been demonstrated to have substantial effects on the migration and regulation of ECM cells (19). However, the prospective impacts of FARGs on immune cell infiltration, HNSCC prognosis, and drug sensitivity have not yet been elucidated. Therefore, We analysed the part FARGs play in the HNSCC prognosis. We built a risk prognosis model determined by FARGs and separated individuals into groups with high and low risks depending on the danger score to forecast the prognosis of the patients. According to results of the calibration diagram and ROC curve, the prognostic model had a good prediction capacity. We screened nine FARGs associated with HNSCC prognosis. Except for PRKCB and PIK3R3 which were potentially protective genes, the remaining seven were potential risk genes. These genes have been linked to cancer initiation and progression. RT-PCR also confirmed that the mRNA levels of ACTB, MAP2K1, MAPK9, PARVB, PDGFA, and PIK3R3 were relatively high in HNSCC tissues. The proteins encoded by MAPK9 and MAP2K1 are mitogen-activated protein (MAP) kinases. MAP kinases can integrate a variety of biochemical signals and participate in cell proliferation, differentiation, transcriptional regulation and development (20). In the research of Ren et al., patients with HNSCC might benefit from using MAPK9 as a separate and trustworthy prognostic marker along with a therapeutic target (21). We knocked down MAPK9 in HNSCC cell lines to further explore and verify biologic function and mechanism of activity of MAPK9 in HNSCC. The outcomes showed that MAPK9 accelerated the metastasis of HNSCC cells by affecting EMT pathway-related proteins, thus verifying the poor prognostic role of MAPK9 in HNSCC. PDGFA, the core gene with increased expression on chromosome 7, widely exists in glioblastomas and has been demonstrated to be crucial in glioma (22). In addition, Cui et al. discovered that colorectal cancer and oesophageal squamous cell carcinoma both exhibit high levels of PDGFA expression (23, 24). ACTB, a cytoskeletal actin, has long been considered a common reference gene for a long time (25). However, increasing evidence has recently shown that cytoskeletal actin can regulate the adhesion and movement of tumour cells, and ACTB can influence the invasion and metastasis of tumour cells via polymerisation and localization (26). ACTB plays important roles in renal cell carcinoma, colorectal cancer, liver cancer etc (27–29). The G1 cycle of cells is regulated by the protein CCND1; therefore, this is considered carcinogenic gene (30). In the study by Liu et al., they indicated that CCND1 amplification is related to the adverse reaction of ICIs, and may contribute to the promotion of tumour immune escape through TGF-β and KRAS signalling pathways (31). THBS1 is a secretory protein that binds to different cellular receptors. It acts on the tumour microenvironment (TME), inhibits angiogenesis, stimulates tumor cell migration, and regulates immune cell infiltration. Previous research has demonstrated that THBS1 protein is highly expressed in lymphoma, stomach cancer, melanoma, breast cancer and HNSCC, and that it promotes the adhesion, growth, metastasis, and invasion of tumor cells (32–35). PARVB has an essential part in actin reorganisation and the formation of focal adhesions and is heavily present in the spleen, skeletal muscle, kidney, and heart, where it can affect cell migration and wound healing. Significantly worse survival of cancer patients with higher parvb expression was found in an investigation by Eslami et al. (36). Nevertheless, it is still unknown how PARV-B causes cancer and requires further exploration. The protein kinase C family member PPKCB is crucial for regulating cell survival and apoptosis (37). Multiple studies have suggested that PPKCB is hypermethylated in a variety of adenocarcinomas, and its expression in tumour cells may be regulated by the Wnt signaling pathway (38). PIK3R3, also known as p55, is a lipid kinase that phosphorylates phosphatidylinositol and similar compounds to produce important secondary messengers in growth-signalling pathways. Ibrahim et al. discovered that colorectal cancer tissues have significantly higher levels of PIK3R3 expression (39). Moreover, several studies have previously reported elevated PIK3R3 expression in multiple malignancies (40, 41).

New therapeutic approaches for HNSCC are currently under investigation. Immunotherapy has emerged as one of the most common anti-tumor therapies in recent years. Individuals with recurrent or metastatic (R/M) HNSCC have shown improved rates of survival using T cell-based immunotherapies (such as ICI) (42). Among them, inhibitors of programmed death receptor-1 (PD-1) and programmed death ligand-1 (PD-L1) exhibit strong anti-tumor activity and are safe for the management of HNSCC. However, not all patients react well to PD-1/PD-L1 immunosuppressants, and the overall rate of response to PD-1/PD-L1 inhibitors in R/M HNSCC patients is less than 20% (43). Additionally, immunotherapy has adverse reactions and drug resistance in clinical applications. Multiple researches have shown that the biological characteristics, dynamic inhibitory changes, and heterogeneity of the TME may determine the effect of immunotherapy (44). Tumour immune cell invasion, which is a significant part of the TME, plays a key role in immunotherapy (45). For example, tumour-associated macrophages (TAM) can enhance immunosuppression through transforming growth factor-β (TGF-β), secreting interleukin-10 (IL-10) etc., which is related to poor prognosis (46). However, increased quantities of tumour-infiltrating lymphocytes (TLSs, CD4+T cells, and CD8+T cells) are related to a good prognosis (47). However, the mechanism underlying immune cell infiltration in HNSCC is not fully understood yet. Although immunotherapy is currently a new treatment option, it is used more as an auxiliary means of personalised treatment according to patient characteristics. Therefore, analysing the therapeutic significance of immune cell infiltration in HNSCC and combining immunotherapy with traditional tumour treatment methods, such as radiotherapy or chemotherapy, to achieve precise and personalised treatment, is currently a clinical problem that needs to be solved.

Studies have been conducted to correlate tumour prognostic markers with tumour immunotherapy and immune infiltration. In Kuang et al. Immunogenic cell death (ICD) is a specific mode of cell death that initiates the host anti-tumor immune response (48). They used the ICD as a tumour prognostic marker for glioma and using the CIBERSORT and ESTIMATE algorithms to assess the variations in tumour purity, immune score, and stromal score of the two ICD subtypes in the tumour microenvironment (TME). Ultimately, the high ICD subgroup had higher immune scores, lower tumour purity, and more active tumour immune responses than the low ICD subgroup, all of which can be used to guide individualised immunotherapy for glioma (49). Cao et al. performed a similar analysis. In their study, long-stranded non-coding RNAs (IncRNAs) were used as glioma In their study, long-stranded non-coding RNAs (IncRNAs) were used as relevant prognostic markers for glioma, and Glioma samples were split into groups with high and low immune cell infiltration using ssGSEA, to analyze stromal score, immune score and ESTIMATE score, and to screen IncRNAs differentially expressed in high and low immune cell infiltration groups. This was done to determine the connection between the lncRNAs and the glioma immune response and immune infiltration of lncRNAs as potential immunotherapeutic targets for glioma (50).

It has recently been shown that focal adhesion kinase (FAK) recruits immune cells, promotes angiogenesis and ECM cell proliferation to drive tumor progression. Serrels et al. showed that FAK recruits Tregs cells to eliminate CD8^+^ T cells by increasing the expression of CCL1, CCL5, CCL7, CXCL10 and TGFβ2, which allows tumour cells to achieve immune escape (51). Li et al. studied the correlation between gliomas and FARGs and found that they were closely associated with tumour prognosis and immune infiltration, which is consistent with our results in HNSCC (52). To further explore the influence of FARGs on immune invasion, we looked into the relationship between immunological invasion and the nine FARGs. Because of rapid advancements in tumour treatment and microarray sequencing, the ESTIMATE algorithm is widely used in the analysis of immune infiltration and the exploration of new targets for immunotherapy (53). In this study, we obtained estimated immune/stromal scores and tumour purity in HNSCC cells using the ESTIMATE algorithm and comprehensively analysed high-throughput data. The findings revealed that the two groups’ scores and tumour purity varied considerably and that the FARG risk score had a significant negative correlation with the estimated score and a significant positive correlation with tumour purity. Moreover, different immune, stromal, and estimated scores and tumour purity significantly affected survival. Additionally, comparing the high-risk and low-risk groups, there were notable variations in the degree of invasion of M2 macrophages, CD8+ T cells, B cells, and myeloid dendritic cells. To directly evaluate the clinical significance of FARGs in immunotherapy, we applied the TIDE algorithm to forecast immunotherapy outcomes. These findings indicate that compared to the low-risk group, the high-risk group’s TIDE score was much lower, showing that immunotherapy works better in the high-risk group. We compared TCGA-HNSCC expression profiles with those of a sample of melanoma patients undergoing immunotherapy using the submap algorithm and showed that the likelihood of an immunological response was higher in the high-risk group. This further validates our conclusion, and we believe that the FARG prognostic signature can provide guidance for tumour immunotherapy.

In addition to exploring the correlation between FARGs and immunotherapy, the sensitivity of the high-risk and low-risk groups were compared to nine popular HNSCC chemotherapy agents. The low-risk group responded to cisplatin, methotrexate, rapamycin, paclitaxel, and mitomycin C with greater sensitivity. The high-risk group showed greater sensitivity to docetaxel, lapatinib, gemcitabine, and bleomycin therapy. Improving the curative effect of individual precision treatment for HNSCC by combining immunotherapy with traditional tumour treatment methods such as radiation or chemotherapy is the current clinical direction. Based on the prediction of immunotherapy and chemotherapy responses in HNSCC patients based on the nine FARG signatures, we can apply more sensitive and effective treatment options to patients, which has great clinical implications in the future.

Moreover, we performed gene mutation analysis of HNSCC. We discovered that the number of mutations was significantly higher in the high-risk group than in the low-risk group by comparing the two groups. Missense mutations and TP53 were the most common types of mutations and mutated genes, respectively. Previous studies revealed that the gene TP53 is the most usually altered in human cancer, including HNSCC, which is consistent with our conclusion (54).

However, this research had certain restrictions. First, more cellular and molecular biology data are required as our research primarily leaned on data from publicly available databases. However, there was a lack of an explanation and examination of the major pathways involved in FARGs.

## 5 Conclusion

In this study, nine FARGs (ACTB, CCND, MAP2K1, MAPK9, PARVB, PDGFA, THBS1, PRKCB, and PIK3R3) were associated with HNSCC prognosis, and based on them, a prognostic risk model was developed. We investigated the immune infiltration, immunotherapy prediction, and drug sensitivity using the constructed model. Finally, we performed a preliminary verification of the expression of these FARGs using molecular biology. In summary, this study provides new perspectives and directions for the diagnosis, treatment, and prognostic prediction of HNSCC.

## Data availability statement

The original contributions presented in the study are included in the article/**Supplementary Material**. Further inquiries can be directed to the corresponding authors.

## Ethics statement

The studies involving humans were approved by the medical ethics committees of The First Affiliated Hospital of Anhui Medical University (Reference number: Quick-PJ 2022-03-19). The studies were conducted in accordance with the local legislation and institutional requirements. The participants provided their written informed consent to participate in this study.

## Author contributions

YCL, ZCW, ZYF, YXH: Conceptualization, methodology, software. JPW, YQZ, BYL: Data curation, writing - original draft preparation. YT, YCZ, CLS, YDX, SYY: Visualization, investigation. BJC, YHL, HFP, ZL, KLW: Supervision. HFP, ZL and KLW as the Corresponding Authors guided the design of the entire experiment and the writing of the paper. YCL, ZCW, ZYF, YXH, JPW, YQZ and BYL were responsible for the data collection, the writing of papers and other scattered work, they contributed equally to this work as first authors. YT, YCZ, CLS, YDX, SYY, BJC and YHL were engaged in the collection of the clinical data. All authors contributed to the article and approved the submitted version.

## Supplementary material

Supplementary Figure 1 We performed and K-M analyses for age, gender, grade, T, N, and stage to determine the relationship between clinical factors and tumor prognosis.

Supplementary Figure 2 The expression of CCND1, PRKCB, and THBS1were not statistically significant.

## References

[1] Johnson DE Burtness B Leemans CR Lui VWY Bauman JE and Grandis JR. Head and neck squamous cell carcinoma. Nat Rev Dis Primers (2020) 6:92. doi: 10.1038/s41572-020-00224-3 33243986 PMC7944998

[2] Sung H Ferlay J Siegel RL Laversanne M Soerjomataram I Jemal A. Global cancer statistics 2020: GLOBOCAN estimates of incidence and mortality worldwide for 36 cancers in 185 countries. CA Cancer J Clin (2021) 71:209–49. doi: 10.3322/caac.21660 33538338

[3] Miyauchi S Kim SS Pang J Gold KA Gutkind JS Califano JA. Immune modulation of head and neck squamous cell carcinoma and the tumor microenvironment by conventional therapeutics. Clin Cancer Res (2019) 25:4211–23. doi: 10.1158/1078-0432.CCR-18-0871 PMC6635035 30814108

[4] Chauhan SS Kaur J Kumar M Matta A Srivastava G Alyass A. Prediction of recurrence-free survival using a protein expression-based risk classifier for head and neck cancer. Oncogenesis (2015) 4:e147. doi: 10.1038/oncsis.2015.7 25893634 PMC4491610

[5] Yokota T Homma A Kiyota N Tahara M Hanai N Asakage T. Immunotherapy for squamous cell carcinoma of the head and neck. Jpn J Clin Oncol (2020) 50:1089–96. doi: 10.1093/jjco/hyaa139 32776100

[6] Gao HX Wang MB Li SJ Niu J Xue J Li J. Identification of hub genes and key pathways associated with peripheral T-cell lymphoma. Curr Med Sci (2020) 40:885–99. doi: 10.1007/s11596-020-2250-9 32980897

[7] Lan Q Wang P Tian S and Dong W. Mining TCGA database for genes of prognostic value in gastric cancer microenvironment. J Cell Mol Med (2020) 24:11120–32. doi: 10.1111/jcmm.15595 PMC7576220 32818296

[8] Nikou S Arbi M Dimitrakopoulos FD Sirinian C Chadla P Pappa I. Integrin-linked kinase (ILK) regulates KRAS, IPP complex and Ras suppressor-1 (RSU1) promoting lung adenocarcinoma progression and poor survival. J Mol Histol (2020) 51:385–400. doi: 10.1007/s10735-020-09888-3 32592097

[9] Kang HR Moon JY Ediriweera MK Song YW Cho M Kasiviswanathan D. Dietary flavonoid myricetin inhibits invasion and migration of radioresistant lung cancer cells (A549-IR) by suppressing MMP-2 and MMP-9 expressions through inhibition of the FAK-ERK signaling pathway. Food Sci Nutr (2020) 8:2059–67. doi: 10.1002/fsn3.1495 PMC7174229 32328272

[10] Li J Hao N Han J Zhang M Li X and Yang N. ZKSCAN3 drives tumor metastasis via integrin beta4/FAK/AKT mediated epithelial-mesenchymal transition in hepatocellular carcinoma. Cancer Cell Int (2020) 20:216. doi: 10.1186/s12935-020-01307-7 32518525 PMC7275473

[11] Eke I Cordes N. Focal adhesion signaling and therapy resistance in cancer. Semin Cancer Biol (2015) 31:65–75. doi: 10.1016/j.semcancer.2014.07.009 25117005

[12] Tumeh PC Harview CL Yearley JH Shintaku IP Taylor EJ Robert L. PD-1 blockade induces responses by inhibiting adaptive immune resistance. Nature (2014) 515:568–71. doi: 10.1038/nature13954 PMC4246418 25428505

[13] Jia Y Liu L and Shan B. Future of immune checkpoint inhibitors: focus on tumor immune microenvironment. Ann Transl Med (2020) 8:1095. doi: 10.21037/atm-20-3735 33145314 PMC7575936

[14] Caudell JJ Gillison ML Maghami E Spencer S Pfister DG Adkins D. NCCN guidelines(R) insights: head and neck cancers, version 1.2022. J Natl Compr Canc Netw (2022) 20:224–34. doi: 10.6004/jnccn.2022.0016 35276673

[15] Posner M Vermorken JB. Induction therapy in the modern era of combined-modality therapy for locally advanced head and neck cancer. Semin Oncol (2008) 35:221–8. doi: 10.1053/j.seminoncol.2008.03.007 18544437

[16] Bray F Ferlay J Soerjomataram I Siegel RL Torre LA and Jemal A. Global cancer statistics 2018: GLOBOCAN estimates of incidence and mortality worldwide for 36 cancers in 185 countries. CA Cancer J Clin (2018) 68:394–424. doi: 10.3322/caac.21492 30207593

[17] Tahara M Muro K Hasegawa Y Chung HC Lin CC Keam B. Pembrolizumab in Asia-Pacific patients with advanced head and neck squamous cell carcinoma: Analyses from KEYNOTE-012. Cancer Sci (2018) 109:771–6. doi: 10.1111/cas.13480 PMC5834807 29284202

[18] Pallasch FB Schumacher U. Angiotensin inhibition, TGF-beta and EMT in cancer. Cancers (Basel) (2020) 12:2785. doi: 10.3390/cancers12102785 32998363 PMC7601465

[19] Murphy JM Rodriguez YAR Jeong K Ahn EE and Lim SS. Targeting focal adhesion kinase in cancer cells and the tumor microenvironment. Exp Mol Med (2020) 52:877–86. doi: 10.1038/s12276-020-0447-4 PMC7338452 32514188

[20] Rato S Maia S Brito PM Resende L Pereira CF Moita C. Novel HIV-1 knockdown targets identified by an enriched kinases/phosphatases shRNA library using a long-term iterative screen in Jurkat T-cells. PloS One (2010) 5:e9276. doi: 10.1371/journal.pone.0009276 20174665 PMC2822867

[21] Ren Z Zhang L Ding W Luo Y Shi Z Shrestha B. Development and validation of a novel survival model for head and neck squamous cell carcinoma based on autophagy-related genes. Genomics (2021) 113:1166–75. doi: 10.1016/j.ygeno.2020.11.017 33227411

[22] Ozawa T Riester M Cheng YK Huse JT Squatrito M Helmy K. Most human non-GCIMP glioblastoma subtypes evolve from a common proneural-like precursor glioma. Cancer Cell (2014) 26:288–300. doi: 10.1016/j.ccr.2014.06.005 25117714 PMC4143139

[23] Han N Zhang YY Zhang ZM Zhang F Zeng TY Zhang YB. High expression of PDGFA predicts poor prognosis of esophageal squamous cell carcinoma. Med (Baltimore) (2021) 100:e25932. doi: 10.1097/MD.0000000000025932 PMC8137088 34011067

[24] Cui HY Wei W Qian MR Tian RF Fu X Li HW. PDGFA-associated protein 1 is a novel target of c-Myc and contributes to colorectal cancer initiation and progression. Cancer Commun (Lond) (2022) 42:750–67. doi: 10.1002/cac2.12322 PMC9395323 35716012

[25] Yang H Zhang L and Liu S. Determination of reference genes for ovine pulmonary adenocarcinoma infected lung tissues using RNA-seq transcriptome profiling. J Virol Methods (2020) 284:113923. doi: 10.1016/j.jviromet.2020.113923 32615131

[26] Guo C Liu S Wang J Sun MZ and Greenaway FT. ACTB in cancer. Clin Chim Acta (2013) 417:39–44. doi: 10.1016/j.cca.2012.12.012 23266771

[27] Nowak D Skwarek-Maruszewska A Zemanek-Zboch M Malicka-Blaszkiewicz M. Beta-actin in human colon adenocarcinoma cell lines with different metastatic potential. Acta Biochim Pol (2005) 52:461–8. 15940343

[28] Ferguson RE Carroll HP Harris A Maher ER Selby PJ and Banks RE. Housekeeping proteins: a preliminary study illustrating some limitations as useful references in protein expression studies. Proteomics (2005) 5:566–71. doi: 10.1002/pmic.200400941 15627964

[29] Gao Q Wang XY Fan J Qiu SJ Zhou J Shi YH. Selection of reference genes for real-time PCR in human hepatocellular carcinoma tissues. J Cancer Res Clin Oncol (2008) 134:979–86. doi: 10.1007/s00432-008-0369-3 PMC12160763 18317805

[30] Malumbres M Barbacid M. To cycle or not to cycle: a critical decision in cancer. Nat Rev Cancer (2001) 1:222–31. doi: 10.1038/35106065 11902577

[31] Liu J Lin J Wang X Zheng X Gao X Huang Y. CCND1 amplification profiling identifies a subtype of melanoma associated with poor survival and an immunosuppressive tumor microenvironment. Front Immunol (2022) 13:725679. doi: 10.3389/fimmu.2022.725679 35844619 PMC9285001

[32] Kamijo H Miyagaki T Takahashi-Shishido N Nakajima R Oka T Suga H. Thrombospondin-1 promotes tumor progression in cutaneous T-cell lymphoma via CD47. Leukemia (2020) 34:845–56. doi: 10.1038/s41375-019-0622-6 31712778

[33] Pal SK Nguyen CT Morita KI Miki Y Kayamori K Yamaguchi A. THBS1 is induced by TGFB1 in the cancer stroma and promotes invasion of oral squamous cell carcinoma. J Oral Pathol Med (2016) 45:730–9. doi: 10.1111/jop.12430 26850833

[34] Jayachandran A Anaka M Prithviraj P Hudson C McKeown SJ Lo PH. Thrombospondin 1 promotes an aggressive phenotype through epithelial-to-mesenchymal transition in human melanoma. Oncotarget (2014) 5:5782–97. doi: 10.18632/oncotarget.2164 PMC4170613 25051363

[35] Zhang X Huang T Li Y and Qiu H. Upregulation of THBS1 is related to immunity and chemotherapy resistance in gastric cancer. Int J Gen Med (2021) 14:4945–57. doi: 10.2147/IJGM.S329208 PMC8407783 34475782

[36] Eslami A Miyaguchi K Mogushi K Watanabe H Okada N Shibuya H. PARVB overexpression increases cell migration capability and defines high risk for endophytic growth and metastasis in tongue squamous cell carcinoma. Br J Cancer (2015) 112:338–44. doi: 10.1038/bjc.2014.590 PMC4453450 25422907

[37] Roffey J Rosse C Linch M Hibbert A McDonald NQ and Parker PJ. Protein kinase C intervention: the state of play. Curr Opin Cell Biol (2009) 21:268–79. doi: 10.1016/j.ceb.2009.01.019 19233632

[38] Liu S Chen X Chen R Wang J Zhu G Jiang J. Diagnostic role of Wnt pathway gene promoter methylation in non small cell lung cancer. Oncotarget (2017) 8:36354–67. doi: 10.18632/oncotarget.16754 PMC5482660 28422739

[39] Ibrahim S Zhu X Luo X Feng Y and Wang J. PIK3R3 regulates ZO-1 expression through the NF-kB pathway in inflammatory bowel disease. Int Immunopharmacol (2020) 85:106610. doi: 10.1016/j.intimp.2020.106610 32473571

[40] Zhou J Kang N Cui L Ba D and He W. Anti-gammadelta TCR antibody-expanded gammadelta T cells: a better choice for the adoptive immunotherapy of lymphoid Malignancies. Cell Mol Immunol (2012) 9:34–44. doi: 10.1038/cmi.2011.16 21666706 PMC4002925

[41] Zhang L Huang J Yang N Greshock J Liang S Hasegawa K. Integrative genomic analysis of phosphatidylinositol 3’-kinase family identifies PIK3R3 as a potential therapeutic target in epithelial ovarian cancer. Clin Cancer Res (2007) 13:5314–21. doi: 10.1158/1078-0432.CCR-06-2660 17875760

[42] Lee MY Allen CT. Mechanisms of resistance to T cell-based immunotherapy in head and neck cancer. Head Neck (2020) 42:2722–33. doi: 10.1002/hed.26158 32275098

[43] Borel C Jung AC and Burgy M. Immunotherapy breakthroughs in the treatment of recurrent or metastatic head and neck squamous cell carcinoma. Cancers (Basel) (2020) 12:2691. doi: 10.3390/cancers12092691 32967162 PMC7563963

[44] Galon J Bruni D. Approaches to treat immune hot, altered and cold tumours with combination immunotherapies. Nat Rev Drug Discovery (2019) 18:197–218. doi: 10.1038/s41573-018-0007-y 30610226

[45] Puram SV Tirosh I Parikh AS Patel AP Yizhak K Gillespie S. Single-cell transcriptomic analysis of primary and metastatic tumor ecosystems in head and neck cancer. Cell (2017) 171:1611–1624 e1624. doi: 10.1016/j.cell.2017.10.044 29198524 PMC5878932

[46] Noy R Pollard JW. Tumor-associated macrophages: from mechanisms to therapy. Immunity (2014) 41:49–61. doi: 10.1016/j.immuni.2014.06.010 25035953 PMC4137410

[47] Vassilakopoulou M Avgeris M Velcheti V Kotoula V Rampias T Chatzopoulos K. Evaluation of PD-L1 expression and associated tumor-infiltrating lymphocytes in laryngeal squamous cell carcinoma. Clin Cancer Res (2016) 22:704–13. doi: 10.1158/1078-0432.CCR-15-1543 26408403

[48] Ahmed A Tait SWG. Targeting immunogenic cell death in cancer. Mol Oncol (2020) 14:2994–3006. doi: 10.1002/1878-0261.12851 33179413 PMC7718954

[49] Kuang Y Jiang B Zhu H Zhou Y Huang H Li C. Classification related to immunogenic cell death predicts prognosis, immune microenvironment characteristics, and response to immunotherapy in lower-grade gliomas. Front Immunol (2023) 14:1102094. doi: 10.3389/fimmu.2023.1102094 37153540 PMC10154552

[50] Cao Y Zhu H Tan J Yin W Zhou Q Xin Z. Development of an immune-related lncRNA prognostic signature for glioma. Front Genet (2021) 12:678436. doi: 10.3389/fgene.2021.678436 34194477 PMC8238205

[51] Serrels A Lund T Serrels B Byron A McPherson RC von Kriegsheim A. Nuclear FAK controls chemokine transcription, Tregs, and evasion of anti-tumor immunity. Cell (2015) 163:160–73. doi: 10.1016/j.cell.2015.09.001 PMC4597190 26406376

[52] Li H Wang G Wang W Pan J Zhou H Han X. A focal adhesion-related gene signature predicts prognosis in glioma and correlates with radiation response and immune microenvironment. Front Oncol (2021) 11:698278. doi: 10.3389/fonc.2021.698278 34631528 PMC8493301

[53] Pan L Fang J Chen MY Zhai ST Zhang B Jiang ZY. Promising key genes associated with tumor microenvironments and prognosis of hepatocellular carcinoma. World J Gastroenterol (2020) 26:789–803. doi: 10.3748/wjg.v26.i8.789 32148377 PMC7052538

[54] Hainaut P Pfeifer GP. Somatic TP53 mutations in the era of genome sequencing. Cold Spring Harb Perspect Med (2016) 6:a026179. doi: 10.1101/cshperspect.a026179 27503997 PMC5088513

