# Supplementary figures and images for: A predictive model of immune infiltration and prognosis of head and neck squamous cell carcinoma based on cell adhesion-related genes: including molecular biological validation
Source: Front Immunol. 2023 Aug 24;14:1190678. doi: 10.3389/fimmu.2023.1190678 (PMC10484396; doi:10.3389/fimmu.2023.1190678)

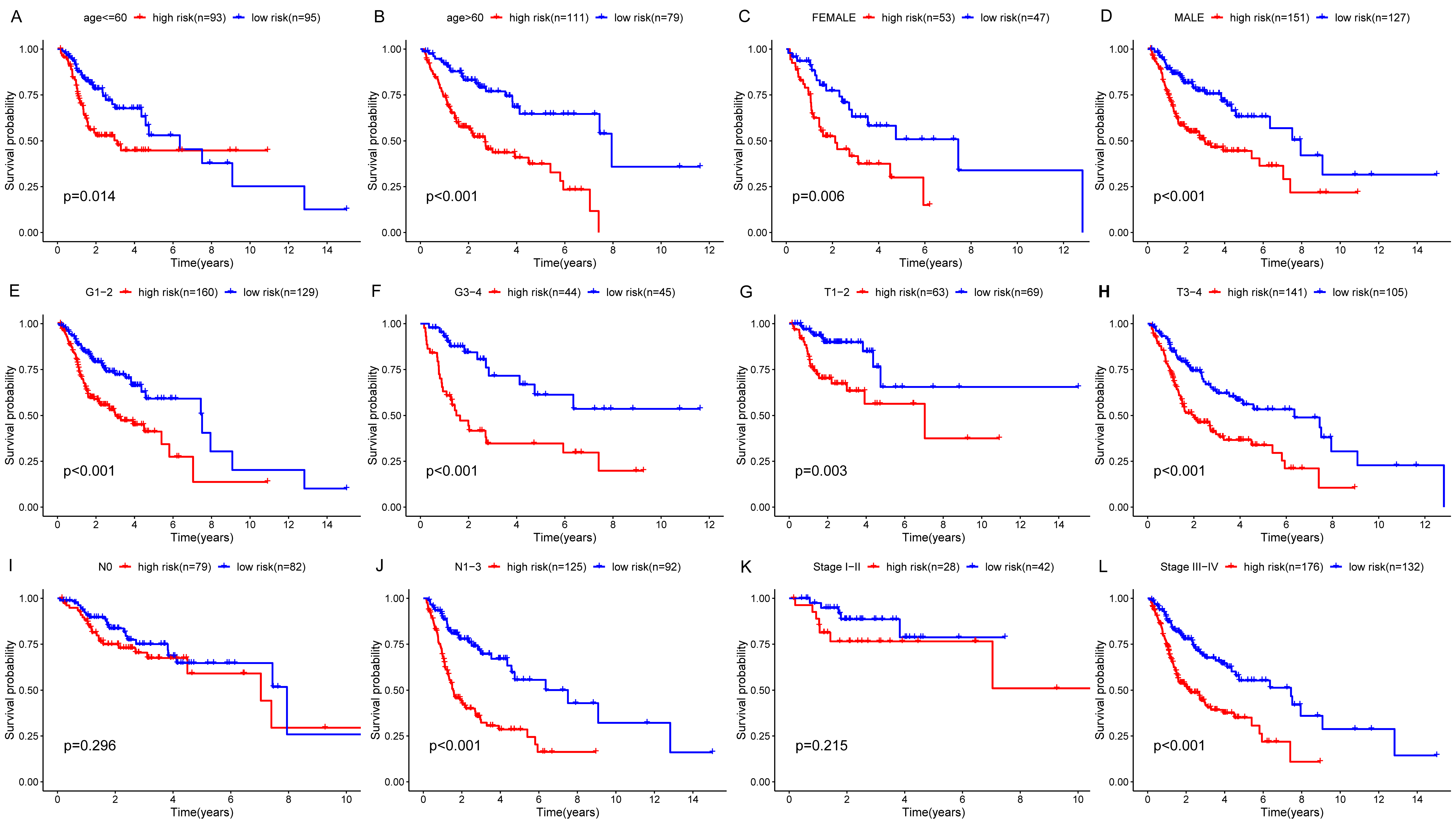

Supplement: Supplementary Figure 1 — We performed and K-M analyses for age, gender, grade, T, N, and stage to determine the relationship between clinical factors and tumor prognosis. [file Image_1.tif]

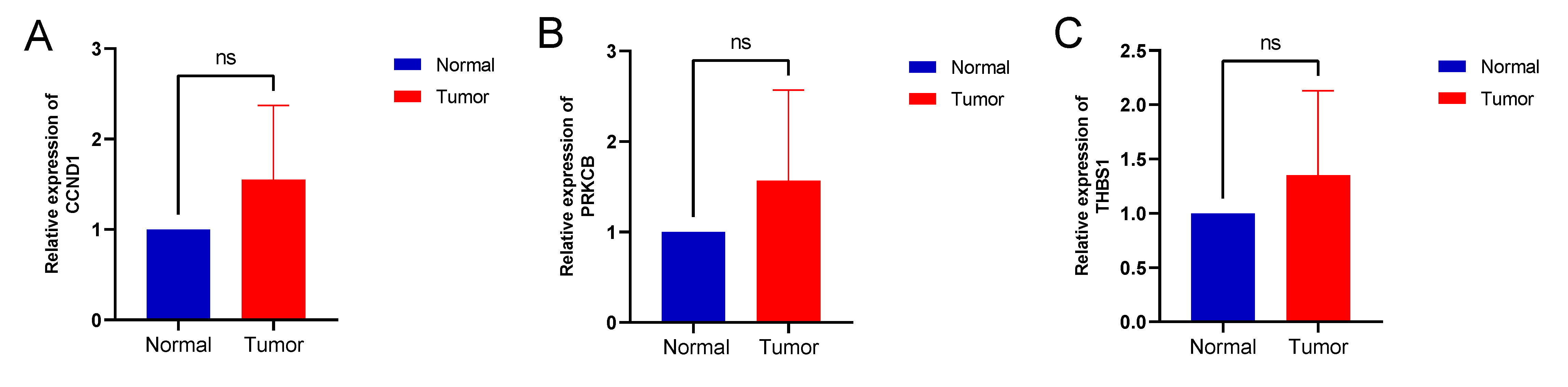

Supplement: Supplementary Figure 2 — The expression of CCND1, PRKCB, and THBS1were not statistically significant. [file Image_2.tif]
