# Supplementary material for: A predictive model of immune infiltration and prognosis of head and neck squamous cell carcinoma based on cell adhesion-related genes: including molecular biological validation
Source: Front Immunol. 2023 Aug 24;14:1190678. doi: 10.3389/fimmu.2023.1190678 (PMC10484396; doi:10.3389/fimmu.2023.1190678)
Supplement: Supplementary file 4 [file Table_2.docx]

**Supplement Table 2.** **The primers used for real-time PCR assays.**

|  | **Forward Primer** | **Reverse Primer** |
| --- | --- | --- |
| **GAPDH** | 5′-GGAGCGAGATCCCTCCAAAAT-3′ | 5′-GGCTGTTGTCATACTTCTCATGG -3′ |
| **ACTB** | 5′- AGCGAGCATCCCCCAAAGTT-3′ | 5′-GGGCACGAAGGCTCATCATT-3′ |
| **CCND1** | 5ʹ-GGAGCCTATTCTGCCCATTT-3′ | 5ʹ-CGAGGTCATAGTTCCTGTTGGTG-3′ |
| **MAP2K1** | 5′-AGCGGATCCCCCGGGTCCAAAATGCCC-3′ | 5′-CTTCTCGAGCACTTAGACGCCAGCAGC-3′ |
| **MAPK9** | 5’-ACCCTTCGGGATATTGCAGG-3’ | 5’-TGCAGCACAAACAATCCCTTG-3’ |
| **PRKCB** | 5′-CGTCCTCATTGTCCTCGTAA-3′ | 5′-TGTCTCATTCCACTCAGGGTT-3′ |
| **PARVB** | 5′-CATCCGCCTTCCTGAGCAT-3′ | 5′-AGCAGGCCTTCCCGTTTC-3′ |
| **PDGFA** | 5′-TGGCCAAGGTGGAATACGTC-3′ | 5′-AGACCGCACACTGGCAATAA-3′ |
| **PIK3R3** | 5′-ATGTACAATACGGTGTGGAGTATG-3′ | 5′-GCTGGAGGATCCATTTCAAT-3′ |
| **THBS1** | 5′-AACGACGGCCAGTGAATTCTATGCTGGTGGTAGACTAGGGTTG-3′ | 5′-GTCATCCTTGTAATCGGGATCTGTTAAATAAAC-3′ |
