# Supplementary material for: A predictive model of immune infiltration and prognosis of head and neck squamous cell carcinoma based on cell adhesion-related genes: including molecular biological validation
Source: Front Immunol. 2023 Aug 24;14:1190678. doi: 10.3389/fimmu.2023.1190678 (PMC10484396; doi:10.3389/fimmu.2023.1190678)
Supplement: Supplementary file 5 [file Table_3.docx]

**Supplement Table 3. Oligonucleotide sequences of MAPK9.**

| **Note** | **Sequences ( 5'-3' )** |
| --- | --- |
| (human) shSc | TTCTCCGAACGTGTCACGT |
| (human) shMAPK9-1 | TGGAATTAATGGATGCTAA |
| (human) shMAPK9-2 | CCGAAGTCATCCTGGGTAT |
